# Cytotoxicity and Mitochondrial Effects of Phenolic and Quinone-Based Mitochondria-Targeted and Untargeted Antioxidants on Human Neuronal and Hepatic Cell Lines: A Comparative Analysis

**DOI:** 10.3390/biom11111605

**Published:** 2021-10-29

**Authors:** Carlos Fernandes, Afonso J. C. Videira, Caroline D. Veloso, Sofia Benfeito, Pedro Soares, João D. Martins, Beatriz Gonçalves, José F. S. Duarte, António M. S. Santos, Paulo J. Oliveira, Fernanda Borges, José Teixeira, Filomena S. G. Silva

**Affiliations:** 1 Mitotag, Biocant Park, Parque Tecnológico de Cantanhede, Núcleo 04, Lote 4, 3060-197 Cantanhede, Portugal; afonsovideira@mitotag.com (A.J.C.V.); velosocaroline@mitotag.com (C.D.V.); jdgmartins@gmail.com (J.D.M.); beatrizgoncalveess@gmail.com (B.G.); duartefeliciano@mitotag.com (J.F.S.D.); antoniomiguel@mitotag.com (A.M.S.S.); jose_8_teixeira@hotmail.com (J.T.); 2 CIQUP/Department of Chemistry and Biochemistry, Faculty of Sciences, University of Porto, 4169-007 Porto, Portugal; sofia_benfeito@hotmail.com (S.B.); pedro_hgsoares@hotmail.com (P.S.); fborges@fc.up.pt (F.B.); 3 CNC—Center for Neuroscience and Cell Biology, CIBB—Center for Innovative Biomedicine and Biotechnology, University of Coimbra, 3004-504 Coimbra, Portugal; pauloliv@cnc.uc.pt

**Keywords:** phenolic-based mitochondriotropic antioxidants, quinone-based mitochondriotropic antioxidants, non-targeted antioxidants

## Abstract

Mitochondriotropic antioxidants (MC_3_, MC_6.2_, MC_4_ and MC_7.2_) based on dietary antioxidants and analogs (caffeic, hydrocaffeic, trihydroxyphenylpropanoic and trihydroxycinnamic acids) were developed. In this study, we evaluate and compare the cytotoxicity profile of novel mitochondria-targeted molecules (generally known as MitoCINs) on human HepG2 and differentiated SH-SY5Y cells with the quinone-based mitochondria-targeted antioxidants MitoQ and SkQ_1_ and with two non-targeted antioxidants, resveratrol and coenzyme Q_10_ (CoQ_10_). We further evaluate their effects on mitochondrial membrane potential, cellular oxygen consumption and extracellular acidification rates. Overall, MitoCINs derivatives reduced cell viability at concentrations about six times higher than those observed with MitoQ and SkQ1. A toxicity ranking for both cell lines was produced: MC_4_ < MC_7.2_ < MC_3_ < MC_6.2_. These results suggest that C-6 carbon linker and the presence of a pyrogallol group result in lower cytotoxicity. MC_3_ and MC_6.2_ affected the mitochondrial function more significantly relative to MitoQ, SkQ1, resveratrol and CoQ_10_, while MC_4_ and MC_7.2_ displayed around 100–1000 times less cytotoxicity than SkQ1 and MitoQ. Based on the mitochondrial and cytotoxicity cellular data, MC_4_ and MC_7.2_ are proposed as leads that can be optimized to develop safe drug candidates with therapeutic application in mitochondrial oxidative stress-related diseases.

## 1. Introduction

Mitochondria are subcellular organelles responsible for most cellular adenosine triphosphate (ATP) production through oxidative phosphorylation (OXPHOS) [1]. During normal metabolism, mitochondria regulate reactive oxygen species (ROS) levels and the cell’s redox state through the concerted action of antioxidant defenses. Under pathological conditions, mitochondrial dysfunction leads to increased ROS production and oxidative stress, a process associated with many health-related disorders, including neurodegenerative diseases, non-alcoholic fatty liver disease, cancer, or cardiovascular events, among others [2,3,4].

**Figure 1:**
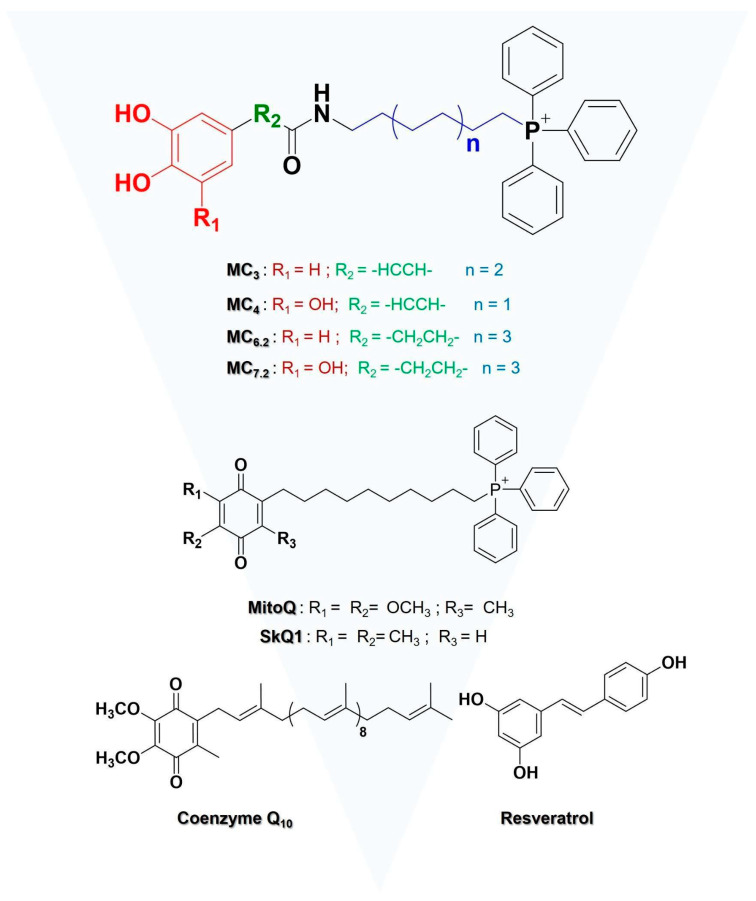
Chemical structures of the mitochondria-targeted (the counterion was omitted for simplicity) and non-targeted antioxidants under study.

Current strategies to decrease excessive mitochondrial ROS production under pathological conditions involve the use of mitochondria-targeted antioxidants. The most popular approach is the conjugation of bioactive molecules to a linker and the lipophilic triphenylphosphonium (TPP^+^) cation [5,6]. MitoQ (with a coenzyme Q core) and SkQ_1_ (with a plastoquinone core) [7,8] are two among the most studied mitochondria-targeted antioxidants (Figure 1). In mitochondria, the quinone moiety is reduced to a quinol, and readily oxidized back to quinone, a process mediated by the respiratory chain, restoring the antioxidant function [8,9,10]. Nevertheless, both MitoQ and SkQ_1_ can also operate as prooxidants, inducing non-specific effects on mitochondria [8,11,12,13].

After promising preclinical studies, the two quinone-based antioxidants entered into clinical trials for diseases such as hepatitis C (NCT00433108), diastolic dysfunction (NCT03586414), Parkinson’s disease (NCT00329056), multiple sclerosis (NCT03166800), treatment of metabolic dysfunction in asthma (NCT04026711), and dry-eye syndrome (NCT03764735 and NCT04206020) (http://clinicaltrials.gov (accessed on 10 September 2021). However, none of the two mitochondria-targeted drug candidates were approved by the Food and Drug Administration (FDA). This may have been related to their failure to reach the primary efficacy endpoints [14]. Thus, new, safer and more effective mitochondria-targeted antioxidants must be developed using other antioxidant cores, such as those of dietary polyphenols. The intake of dietary polyphenols is beneficial due to their antioxidant properties and ability to regulate cellular oxidative stress events. Different mechanisms are proposed for the antioxidant effects of polyphenols: the removal of ROS by direct scavenging of free radicals, upregulation of the activity of ROS-removing enzymes and endogenous antioxidants, chelation of transition metals, and direct inhibition of ROS-producing enzymes [15]. The latter three mechanisms are the most likely to be prevalent physiologically [15].

Among dietary antioxidants, hydroxycinnamic acids (HCAs), such as caffeic or ferulic acids, showed a relevant antioxidant performance in cell-free and cell-based models [16]. However, they have bioavailability constraints, mainly related to their physicochemical properties, including low lipophilicity [17,18,19], and unspecific intracellular accumulation. The latter caveat implies a reduced accumulation on intracellular sites of augmented ROS production, such as mitochondria [20,21,22].

To overcome HCAs’ setbacks, and as an alternative to MitoQ and SkQ_1_, a library of mitochondriotropic antioxidants based on HCAs and its analogs was developed [23,24]. The novel mitochondria-targeted HCA family known as MitoCINs or AntiOxCINs also TPP^+^ as a carrier and showed a higher antioxidant, iron-chelating properties, the capacity to increase glutathione (GSH) concentrations, and similar or higher mitochondrial accumulation capacity when compared with MitoQ [23]. In a previous study, four novel mitochondria-targeted HCAs, namely MC_3_, MC_4_, MC_6.2_ and MC_7.2_ (Figure 1), were described as having unique properties to prevent progressive mitochondrial dysfunction [25].

Extending our previous studies, the novelty in the present work is the comparison of the cytotoxicity and mitochondrial profile of selected MitoCINs library compounds (MC_3_, MC_6.2_, MC_4_ and MC_7.2_) (Figure 1), with the mitochondria-targeted antioxidants MitoQ and SkQ_1_ (Figure 1). Furthermore, we compared the effects of the four MitoCINs molecules with two non-targeted antioxidants that have been tested under clinical trials [26,27,28], namely resveratrol (NCT01914081, NCT03933163) and CoQ_10_ (NCT04780074, NCT04038034) (Figure 1).

Resveratrol is a phenolic compound with antioxidant properties and beneficial effects in age-related diseases [29,30], including neurodegeneration [31]. Although resveratrol can protect neurons from oxidative stress extracellular toxins and insults associated with neurodegenerative disorders through sirtuin 1 activation [32,33,34], it has low mitochondrial accumulation and low bioavailability [35]. Coenzyme Q_10_ (CoQ_10_) has been extensively proposed as a potential therapeutic agent for different neurodegenerative and hepatic diseases; however, its low bioavailability forces it to be administered in substantially high doses, resulting in side-effects [36]. Disappointing results were obtained in some of the clinical trials performed using CoQ_10_ [37,38] and resveratrol [39,40].

For the cytotoxicity comparison of the different classes of compounds, we used HepG2 and differentiated SH-SY5Y cells as in vitro models, since they are often used in the preclinical safety assessment of drug candidates in hepatic and neurodegenerative diseases, respectively [41,42,43]. Although HepG2 is a human hepatoma cell line, it is one of the most commonly used cell models in cytotoxicity screening of different compounds due to its stable phenotype, high availability, easy handling, and phase I and phase II metabolizing activities [44].

The objective of this study was to advance our knowledge of the cytotoxicity of the selected MitoCINs at the mitochondrial level in two different cell systems, compared with two mitochondria-targeted and two untargeted antioxidants, which have been used in clinical trials. The results of our work will contribute to the development of novel and more effective mitochondrial therapies against neurodegenerative and hepatic diseases.

## 2. Material and Methods

### 2.1. Reagents and Cells

All reagents and solvents for chemical synthesis were purchased from Merck Life Science S.L.U (Sintra, Portugal), TCI (Zwijndrecht, Belgium) and Carlo Erba Reactifs (Barcelona, Spain) and used without any further purification. Dulbecco’s Modified Eagle’s Medium (DMEM-D5030), L-Glutamine (G3126), sodium bicarbonate (S6014), sodium pyruvate (P5280), 2-[4-(2-hydroxyethyl)piperazin-1-yl]ethanesulfonic acid (HEPES) (H3375), sulforhodamine B sodium salt (S9012), hydrochloric acid 37% (30721), resazurin sodium salt (R7017), dimethyl sulfoxide (DMSO) (34869), carbonyl cyanide 4-(trifluoromethoxy)phenylhydrazone (FCCP) (C2920), rotenone (R8875) and antimycin A (A8674) were obtained from Merck Life Science S.L.U (Sintra, Portugal). Caffeic acid, hydrocaffeic acid, trihydroxycinnamic acid (TriOH), trihydroxyphenylpropanoic acid (hydro-TriOH), resveratrol (R5010) and CoQ_10_ (C9538) were purchased from Sigma (Sintra, Portugal). Fetal bovine serum (FBS) (10270106), penicillin-streptomycin (15140122), tetramethylrhodamine, methyl ester, perchlorate (TMRM) (T668), Hoechst 33342, Trihydrochloride, Trihydrate (H1399) were obtained from Gibco-Invitrogen (Waltham, MA, USA). From VWR (Amadora, Portugal), we obtained the d-glucose (0188). Glacial acetic acid (A/0400/PB15), methanol (M/4056/17), sodium hydroxide (P/5640/60) were obtained from Fisher Chemical (Porto Salvo, Portugal). Tris base was obtained from ChemCruz (sc-3715) (Heidelberg, Germany). The CellTiter-Glo^®^ Luminescent Cell Viability Assay (PROMG7571) was provided by Promega (Madison, WI, USA). Seahorse XFe96 FluxPak (102416-100) was obtained from Agilent (Santa Clara, CA, USA). Oligomycin (495455) was obtained from Calbiochem (Darmstadt, Germany).

### 2.2. Chemical Synthesis

#### 2.2.1. General Conditions

The progression of the synthetic reactions was followed by thin-layer chromatography (TLC), as previously described by Teixeira et al. [23]. The work-up process for each crude product was performed as described [23,25,45]. The purification of the compounds was performed by flash column chromatography using silica gel 60 (0.040–0.063 mm) and/or recrystallization.

The compound’s structural characterization was produced by ^1^H and ^13^C nuclear magnetic resonance (NMR) spectra, electron impact mass spectra (EI-MS) and electrospray ionization (ESI), in the conditions reported by Benfeito et al. [25].

#### 2.2.2. General Procedure for Synthesis of TPP^+^-Based Antioxidants

The synthetic methodologies and spectroscopic characterization data (Nuclear Magnetic Resonance Spectroscopy and Mass Spectrometry) of the phenolic mitochondriotropic antioxidants MitoCINs (MC_3_, MC_4_, MC_6.2_ and MC_7.2_) were previously described by Benfeito et al. [23,25]; the synthesis of the quinone–based mitochondriotropic antioxidants (MitoQ and SKQ_1_) was based on the procedures reported by James et al. [12] and Korshunova et al. [46].

### 2.3. Cell Culture Conditions and Treatments with Compounds

#### 2.3.1. HepG2 Cells

HepG2 cells were cultured in DMEM (D5030) supplemented with 10% (*v*/*v*) FBS, 1% (*v*/*v*) antibiotic penicillin-streptomycin, 3.7 g/L sodium bicarbonate, 0.876 g/L L-glutamine, 0.11 g/L sodium pyruvate and 0.90 g/L glucose. These cells were also supplemented with 1.19 g/L HEPES. All cells were cultured in monolayer in adherent tissue culture dishes at 37 °C in a humidified atmosphere of 5% CO_2_. Cells were subjected to trypsinization using standard methods when reaching 80–90% confluence and were only used between passages 5 to 25 in cultures in log-phase growth. HepG2 were seeded at 3.0 × 10^4^ cells/cm^2^ for extracellular flux analysis and TMRM assays, and at 6.0 × 10^4^ cells/cm^2^ for the remaining experiments.

#### 2.3.2. Differentiated SH-SY5Y Cells

SH-SY5Y cells were cultured in DMEM (D5030) supplemented with 10% (*v*/*v*) FBS, 1% (*v*/*v*) antibiotic penicillin-streptomycin, 3.7 g/L sodium bicarbonate, 0.876 g/L L-glutamine, 0.11 g/L sodium pyruvate, 4.5 g/L glucose and 1.19 g/L HEPES. Cells were cultured in monolayer in adherent tissue culture dishes at 37 °C in a humidified atmosphere of 5% CO_2_ and were passaged by trypsinization using standard methods when reaching 80–90% confluence, only being used between passages 14 to 25 in cultures in log-phase growth. For the differentiation process, SH-SY5Y cells were seeded (2.4 × 10^4^ cells/cm^2^) with cell culture medium supplemented with retinoic acid (RA) 10 μM for 3 days. On the third day, the cell culture medium was replaced by a fresh one supplemented with 12-o-tetradecanoylphorbol-13-acetate (TPA) 80 nM, allowing the cells to grow in this medium for an additional period of three days before starting the experimental procedure [47].

### 2.4. Compound Treatments

HepG2 cells were seeded for 24 h, during which they reached 40–60% confluence. SH-SY5Y cells were seeded and subjected to the differentiation protocol mentioned above (Section 2.3.2). Cells were then incubated with increasing concentrations of MitoCIN compounds (MC_3_, MC_6.2_, MC_4_, MC_7.2_), the two mitochondria-targeted antioxidants (MitoQ and SkQ_1_) and the two non-targeted antioxidants (resveratrol, CoQ_10_). Stock solutions of all tested compounds at a final concentration of 100 mM were prepared in DMSO, except for CoQ_10_, which was dissolved in chloroform. The maximal concentration of all solvents used in the cellular treatments was 0.1%. As a secondary control, we tested the cytotoxicity of the MitoCIN parentals (caffeic acid, hydrocaffeic acid, TriOH, hydro-TriOH) and the corresponding alkyl-TPP chains (TPP-C6, TPP-C8 and TPP-C10) for 48 h in a low-glucose culture medium. The experiments with MitoCIN compounds, mitochondrial-targeted antioxidants, non-targeted antioxidants, MitoCIN parentals, and alkyl-TPP chains were performed in parallel under the same experimental conditions. Cellular metabolic activity and cell mass were assessed using resazurin reduction and a sulforhodamine B (SRB) assay. Intracellular ATP levels and mitochondrial polarization were determined by commercial fluorescent dyes. Cellular oxygen consumption (OCR) and Extracellular Acidification Rate (ECAR) were measured using a Seahorse XFe96 Extracellular Flux Analyzer.

### 2.5. Cell Mass

Sulforhodamine B was used for the measurement of cellular protein content, which is proportional to cell mass [48]. After 48 h of treatment, the culture medium was removed and the wells were washed with PBS (1X). With 100 μL of 1% (*v*/*v*) acetic acid in methanol, the cells were fixed overnight at −20 °C. After discarding the fixation solution, the plates were dried in an incubator at 37 °C. Next, we added 70 μL of SRB 0.05% (*w*/*v*) and incubated it at 37 °C for 1 h. The wells were washed with 1% (*v*/*v*) of acetic acid in water and dried again. Lastly, 125 μL of 10.5 mM tris-NaOH was used to solubilize the SRB and absorbance was measured at 510 nm, subtracting background measured at 620 nm in the CLARIOstar Plus microplate reader (BMG Labtech, Ortenberg, Germany).

### 2.6. Cell Metabolic Activity

Resazurin reduction assay was used to determine the cells’ metabolic activity [48]. After the incubation time, the culture medium was removed, and the cells were incubated with 10 μg/mL of resazurin solution for 1 h to 1 h 30 at 37 °C and 5% CO_2_. The reduction of resazurin to resorufin was measured fluorometrically at 540 nm excitation and 590 nm emission in the CLARIOstar Plus microplate reader (BMG Labtech, Ortenberg, Germany)

### 2.7. Intracellular ATP Levels

The cells were seeded in 150 μL of cell culture medium in a white opaque bottom 96-well plate for 48 h. After the incubation time, intracellular ATP levels were measured using CellTiter-Glo^®^ Luminescent Cell Viability Assay (Promega, Madison, WI, USA). For that purpose, the culture medium was removed from each well and mixed with an equal amount of the CellTiter-Glo reagent. After that, 100 μL of the mixture was added to each well and, in a microplate reader, the plate was mixed for 2 min to induce cell lysis. After 10 min of incubation, a luminescent signal proportional to the amount of ATP present was generated and measured in a CLARIOstar Plus microplate reader (BMG Labtech, Ortenberg, Germany). The ATP standard curve was generated following the manufacturer’s instructions.

### 2.8. Mitochondrial Polarization

TMRM staining is a fluorescent dye that monitors mitochondrial function and mitochondrial membrane electric potential [49]. TMRM fluorescence signal intensity is proportional to the mitochondrial membrane potential. After cell culture and treatment with the different compounds in black 96-well plates, TMRM was added to each well to provide a final working concentration of 100 nM dye in cell culture medium without FBS. Simultaneously, 1 mg/mL of Hoechst 33342 was added to label the nuclei and to normalize the results. After 25 min of incubation at 37 °C and 5% CO_2_, the cell culture media with TMRM and Hoechst 33342 was removed and replaced by 100 μL of pre-warmed cell culture medium without FBS. The fluorescence signal was recorded using a 548 nm excitation wavelength and a 575 nm emission wavelength in the CLARIOstar Plus microplate reader (BMG Labtech, Ortenberg, Germany).

### 2.9. Cellular Oxygen Consumption and Extracellular Acidification Rates

HepG2 cells were seeded in pre-coated (with collagen I 0.15 mg/mL) Agilent Seahorse XF96 cell culture microplates. SH-SY5Y cells were seeded in the same type of XF96 cell culture microplate without the pre-coating step. The cell densities were as follows: HepG2 1 × 10^4^ cells/80 μL/well; SH-SY5Y 5.5 × 10^3^ cells/103 μL/well. OCR and ECAR were measured at 37 °C using a Seahorse XFe96 Extracellular Flux Analyzer (Agilent Scientific Instruments, Santa Clara, CA, USA).

The day before the experiment, an XFe96 sensor cartridge for each cell plate was placed in a 96 well calibration plate with 200 μL/well of calibration buffer and left to hydrate overnight at 37 °C. On the following day, medium from the plates was replaced with 175 μL/well of pre-warmed, low-buffered, serum-free minimal DMEM medium, supplemented with glucose, glutamine and sodium pyruvate, with pH adjusted to 7.4 and incubated at 37 °C for 1 h to allow the temperature and pH of the medium to reach equilibrium before the first-rate measurement. Oligomycin, FCCP, rotenone (ROT) and antimycin A (AA) were prepared in low-buffered serum-free DMEM medium, supplemented with glucose, glutamine and sodium pyruvate and the pH was adjusted to 7.4. These compounds were loaded into cartridge ports A, B and C (ROT/AA), respectively: HepG2 (2 μM oligomycin, 2 μM FCCP, 1 μM ROT/AA) and SH-SY5Y (2 μM oligomycin, 0.5 μM FCCP, 1 μM ROT/AA). After 1 h of calibration, the 96 well calibration plate was replaced by the cell plate. Oligomycin, FCCP and ROT/AA were then injected by the XFe96 Analyzer into each well at sequential times. The analysis of the OCR-related parameters was performed using the Agilent Seahorse XF Cell Mitostress test report generator software (version 2.6.0).

### 2.10. Data Preprocessing and Statistical Analysis

#### 2.10.1. Data Preprocessing

The data from all the experiments were preprocessed using the spreadsheet program Google sheets and R statistical software.

The data outputs from CLARIOstar Plus microplate reader (BMG Labtech, Ortenberg, Germany) and Seahorse XFe96 Extracellular Flux Analyzer (Agilent Scientific Instruments, Santa Clara, CA, USA) were compiled into standardized data sets where the background signals were subtracted and the cross-plate systematic effects were mitigated by normalizing all the values to a percentage score using the following formula:

$$\mathrm{Percentage}\;\mathrm{of}\;\mathrm{control}\;\left(\%\right)=\frac{t-{\underline{x}}_{0}}{{\underline{x}}_{1}-{\underline{x}}_{0}\;}\times 100$$

where *t* is the absorbance value of the treatment well and ${\underline{x}}_{0}\;\mathrm{and}\;{\underline{x}}_{1}$ are the background and control means, respectively, of the absorbance values of the treatment plate.

All plate replicates per condition were summarized by their mean values to obtain the respective biological observations of the treatments.

Observations outside 1.5 times the interquartile range (IQR) of the sample’s first and third quartiles were considered outliers and not considered for the analysis.

#### 2.10.2. Statistical Analysis

Statistical analysis was performed using R statistical software. The comparisons between groups were performed between each compound and the untreated control or between different compounds, using Welch’s *t*-test. *p*-values were corrected using Bonferroni’s methodology and deemed statistically significant if <0.05.

## 3. Results

### 3.1. Chemistry

The mitochondriotropic antioxidants (MC_3_, MC_4_, MC_6.2_ and MC_7.2_) and their respective intermediates were synthesized according to the method used by Benfeito et al. [23,25], while MitoQ and SkQ1 were prepared based on the procedures described by James et al. [12] and Korshunova et al. [46], respectively. The structural characterization was performed by NMR (^1^H, ^13^C and DEPT) and mass spectroscopy (EI-MS and ESI-MS) techniques, thereby confirming their chemical identity. The desired compounds were obtained in moderate yields (>40%) with a grade of purity higher than 99%, which was determined through high-performance liquid chromatography (data not shown).

### 3.2. Comparative Cytotoxicity between MitoCINs and Targeted- and Non-Targeted Antioxidants

MC_3_, MC_6.2_, MC_4_ and MC_7.2_ are caffeic, hydrocaffeic, trihydroxyphenylpropanoic and trihydroxycinnamic acid-based derivatives linked by an alkyl chain to TPP cation, respectively (Figure 1). Their cytotoxicity was evaluated and compared with MitoQ, SkQ_1_, resveratrol and CoQ_10_ in HepG2 and differentiated SH-SY5Y cells by measuring alterations in cell mass, metabolic viability and intracellular ATP levels. Cells were incubated with the compounds at concentrations between 1–100 μM for 48 h.

**Figure 2:**
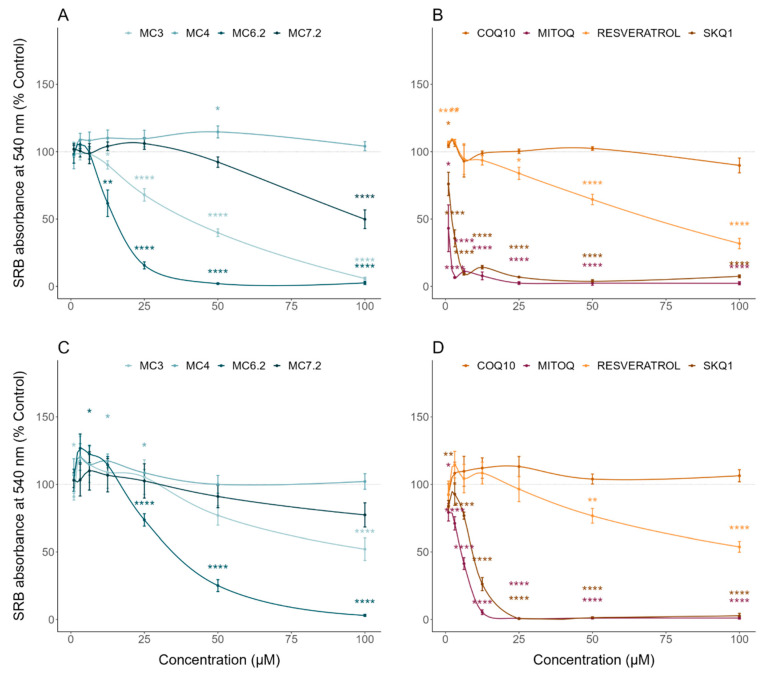
Effect of MitoCINs, quinone-based mitochondria-targeted and non-targeted antioxidants on cell mass. Human Caucasian hepatocyte carcinoma (HepG2, (**A**,**B**)) and differentiated human neuroblastoma (SH-SY5Y, (**C**,**D**)) were treated with increasing concentrations of the different molecules for a period of 48 h and cellular mass was evaluated using Sulforhodamine B (SRB) assay. Data are the mean ± SE of four independent experiments and the results are expressed as a percentage of the control. Statistically significant differences between control (CTL) and treated groups were evaluated as described in the Materials and Methods section. **** *p* < 0.0001, ** *p* < 0.01 and * *p* < 0.05 compared to the respective control (CTL).

**Figure 3:**
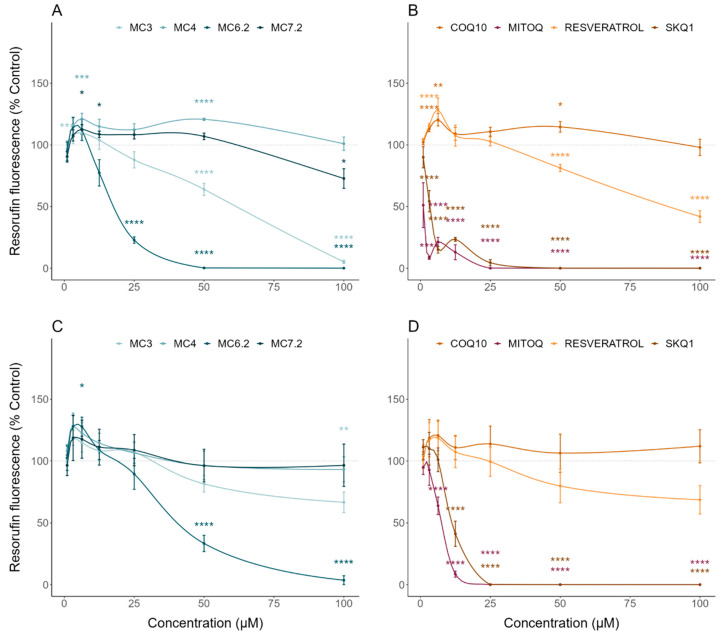
Effect of MitoCINs, quinone-based mitochondria-targeted and non-targeted antioxidants on metabolic activity. Human Caucasian hepatocyte carcinoma (HepG2, (**A**,**B**)) and differentiated human neuroblastoma (SH-SY5Y, (**C**,**D**)) were treated with increasing concentrations of the different molecules for a period of 48 h and metabolic activity was evaluated using resazurin reduction assay. Data are the mean ± SE of four independent experiments and the results are expressed as a percentage of the control (control = 100%). Statistically significant differences between control (CTL) and treated groups were evaluated using a *t*-test. **** *p* < 0.0001, *** *p* < 0.001, ** *p* < 0.01 and * *p* < 0.05 compared to the respective control (CTL).

**Figure 4:**
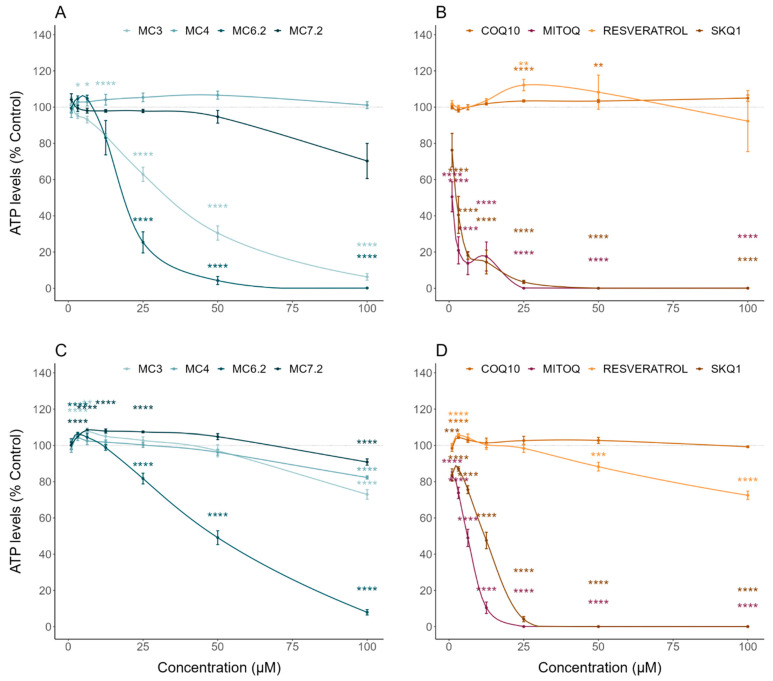
Effect of MitoCINs, quinone-based mitochondria-targeted and non-targeted antioxidants on ATP intracellular concentrations. Human Caucasian hepatocyte carcinoma (HepG2, (**A**,**B**)) and differentiated human neuroblastoma (SH-SY5Y, (**C**,**D**)) were treated with increasing concentrations of the different molecules for a period of 48 h, and intracellular ATP levels were evaluated using CellTiter-Glo^®^ Luminescent Cell Viability Assay. Data are the mean ± SE of three independent experiments and the results are expressed as a percentage of the control (control = 100%). Statistically significant differences between control (CTL) and treated groups were evaluated using a *t*-test. **** *p* < 0.0001, *** *p* < 0.001, ** *p* < 0.01 and * *p* < 0.05 compared to the respective control (CTL).

MC_3_ significantly decreased HepG2 cell mass and metabolic activity at concentrations above 12.5 μM (Figure 2A) and 25 μM (Figure 3A), compared to untreated cells, respectively. In agreement with this, MC_3_ significantly decreased HepG2 ATP levels at concentrations above 12.5 μM compared to untreated cells (Figure 4A). MC_3_ also decreased differentiated SH-SY5Y cell mass (Figure 2C) and metabolic activity (Figure 3C) at concentrations above 100 μM. MC_3_ showed a dual effect in differentiated SH-SY5Y cells, increasing ATP levels at concentrations of 6.3 μM and decreasing them at 100 μM, compared to untreated cells (Figure 4C).

MC_6.2_ significantly decreased HepG2 cell mass at concentrations above 6.3 μM, compared to untreated cells (Figure 2A). Our results also showed that MC_6.2_ decreased cell metabolic activity (Figure 3A) and intracellular ATP levels (Figure 4A) at concentrations above 12.5 μM. In differentiated SH-SY5Y cells, MC_6.2_ showed a dual effect, increasing cell mass at 3.2 μM and decreasing it at 25 μM (Figure 2C). MC_6.2_ also showed a dual effect in differentiated SH-SY5Y cells, increasing their metabolic activity at 6.3 μM and decreasing it at 25 μM (Figure 3C). As observed for MC_3_, MC_6.2_ also increased intracellular ATP levels at 3.2 μM and decreased them at 25 μM (Figure 4C).

No alterations were observed in HepG2 cell mass treated with MC_4_ compared to untreated cells at concentrations up to 100 μM (Figure 2A). Cellular metabolic activity significantly increased at 6.3 μM and 50 μM (Figure 3A). Our results also showed that the cells treated with MC_4_ at concentrations up to 100 μM presented similar values of intracellular ATP levels compared to untreated cells (Figure 4A). Additionally, MC_4_ increased differentiated SH-SY5Y cell mass at 12.5 and 25 μM (Figure 2C), while not affecting the metabolic activity of the differentiated SH-SY5Y cells (Figure 3C). As with the metabolic activity, MC_4_ treatment did not affect the intracellular ATP levels of the differentiated SH-SY5Y cells (Figure 4C).

HepG2 cells treated with MC_7.2_ showed a decrease in cell mass at 100 μM compared to the untreated cells (Figure 2A). On the other hand, cells treated with MC_7.2_ presented decreased metabolic activity at 100 μM (Figure 3A). However, MC_7.2_ did not alter intracellular ATP levels at concentrations up to 100 μM (Figure 4A). In the differentiated SH-SY5Y cells, no alterations were observed in cell mass (Figure 2C) and metabolic activity (Figure 3C) up to 100 μM. Additionally, MC_7.2_ increased intracellular ATP levels at concentrations between 3.2–25 μM and decreased them at 100 μM compared to untreated cells (Figure 4C). Together, these results suggest that MC_4_ and MC_7.2_ presented lower cytotoxicity than MC_3_ and MC_6.2_ in both HepG2 and differentiated SH-SY5Y cells.

To obtain further information on the cytotoxicity mechanisms of the four MitoCIN molecules, we also measured the cytotoxicity of the parental compounds of all four MitoCINs, as well as their alkylTPP constituents.

The caffeic, hydrocaffeic acid, hydro-TriOH and TriOH parental compounds, up to 100 μM, did not cause any alterations in HepG2 cell mass (Figure S1A). Additionally, the HepG2 cells treated with caffeic acid, hydrocaffeic acid and hydro-TriOH showed higher metabolic activity compared to the untreated cells (caffeic acid: 6.3, 25 and 50 μM, hydrocaffeic acid: 6.3 and 50 μM and hydro-TriOH: 3.2, 6.3, 25 and 50 μM), while TriOH did not affect the metabolic activity (Figure S2A). Additionally, hydrocaffeic acid increased HepG2 ATP levels at concentrations of 25 and 100 μM. However, no alterations in ATP levels were observed in the HepG2 cells treated with caffeic acid, TriOH and HydroTriOH at concentrations up to 100 μM (Figure S3A). Similarly, from the data attained on HepG2 cells, no alterations were observed in the differentiated SH-SY5Y cell mass when treated with caffeic, hydroTriOH and TriOH at concentrations up to 100 μM (Figure S1C), although hydrocaffeic acid differentiated SH-SY5Y at 12.5 μM compared to untreated cells (Figure S1C). No alterations in metabolic activity were observed in the differentiated SH-SY5Y cells treated with caffeic acid, hydrocaffeic acid and hydro-TriOH at concentrations up to 100 μM, unlike TriOH, which reduced metabolic activity at 100 μM (Figure S2C). Caffeic acid did not affect intracellular ATP levels in the differentiated SH-SY5Y cells, while hydrocaffeic acid (6.3 and 25 μM) and hydro-TriOH (12.5 μM) increased ATP levels. By contrast, TriOH decreased ATP levels in the differentiated SH-SY5Y cells at 1 μM and at concentrations higher than 25 μM compared to the untreated cells (Figure S3C). Comparing MitoCINs with their respective parental compounds, we observed that MC_3_, MC_6.2_ and MC_7.2_ presented higher cytotoxicity than caffeic, hydrocaffeic acid and hydro TriOH, respectively, in both HepG2 and differentiated SH-SY5Y cells, while MC_4_ presented a similar cytotoxicity compared to the parental TriOH (Figures S4B,D, S5B,D and S6B,D).

Relative to the alkylTPP controls, both TPP-C8 and TPP-C10 reduced HepG2 cell mass (Figure S1B), metabolic activity (Figure S2B) and intracellular ATP levels (Figure S3B) at 1 μM. TPP-C6 at concentrations above 1 μM decreased HepG2 cell mass (Figure S1B), metabolic activity (Figure S2B) and ATP levels (Figure S3B). The differentiated SH-SY5Y cells were less susceptible to the cytotoxicity of alkylTPP derivatives; TPP-C6 (25 μM), TPP-C8 (25 μM) and TPP-C10 (1 μM) reduced cell mass, while TPP-C6 (12.5 and 50 μM), TPP-C8 (3.2 and 50 μM) and TPP-C10 (1 μM) treatment resulted in lower ATP levels (Figure S3D) and reduced metabolic activity (Figure S2D). In general, our results show that MC_3_, MC_6.2_, MC_4_ and MC_7.2_ presented lower cytotoxicity than TPP-C6, TPP-C8 and TPP-C10 in both HepG2 and differentiated SH-SY5Y cells (Figures S4B,D, S5B,D and S6B,D).

By analyzing the cytotoxic effects of the other type of mitochondria-targeted antioxidants, our results demonstrate that HepG2 cells treated with MitoQ and SkQ_1_ showed decreased cell mass (Figure 2B) and metabolic activity (Figure 3B) at concentrations above 3.2 μM compared to untreated cells. MitoQ and SkQ_1_ also decreased ATP levels of HepG2 cells at concentrations above 3.2 and 1 μM, respectively (Figure 4B). In the differentiated SH-SY5Y cells, MitoQ and SkQ_1_ reduced cell mass (Figure 2D) and metabolic activity (Figure 3D) at concentrations above 3.2 μM. The differentiated SH-SY5Y cells treated with MitoQ and SkQ_1_ also presented lower ATP levels at concentrations above 1 μM compared to untreated cells (Figure 4D).

Comparing the cytotoxic effect of MC_3_ with that of the two quinone-based mitochondrial-targeted antioxidants, we found a higher reduction of cell mass and intracellular ATP levels of HepG2 cells treated with MitoQ and SkQ_1_ relative to MC_3_ at concentrations above 1 μM (Figures S4A and S6A). Additionally, the cells treated with MitoQ and SkQ_1_ already showed decreased metabolic activity compared MC_3_ at 3.2 μM (Figure S5A). A superior reduction in differentiated SH-SY5Y cell mass and metabolic activity was observed after MitoQ and SkQ_1_ treatment compared to MC_3_ above 3.2 and 6.3 μM, respectively (Figures S4C and S5C). The cells treated with MitoQ and SkQ_1_ at concentrations above 1 μM showed a decrease of ATP values compared to cells treated with identical concentrations of MC_3_ (Figure S6C). These results suggest that MC_3_ presented lower cytotoxicity than mitochondrial-targeted antioxidants MitoQ, SkQ_1_ in HepG2 and SH-SY5Y cells.

Similarly to MC_3_, the cells treated with MC_6.2_ presented a lower reduction in cell mass, metabolic activity and intracellular ATP levels compared to MitoQ and SkQ_1_ at concentrations higher than 1 μM (Figures S4A, S5A and S6A). The differentiated SH-SY5Y cells treated with MitoQ and SkQ_1_ presented a significantly higher reduction of cell mass compared to MC_6.2_ at concentrations higher than 1 and 3.2 μM, respectively (Figure S4C). The cells treated with MitoQ and SkQ_1_ showed a higher loss of metabolic activity than MC_6.2_ at concentrations higher than 3.2 and 6.3 μM, respectively (Figure S5C). Additionally, the cells treated with MitoQ and SkQ_1_ at concentrations above 1 μM showed lower ATP values compared to cells treated with identical concentrations of MC_6.2_ (Figure S6C). These results also suggest that MC_6.2_ is less toxic than the mitochondria-targeted antioxidants MitoQ and SkQ_1_ in the two cell lines.

HepG2 cells treated with MitoQ and SkQ_1_ at concentrations above 1 μM presented significantly lower cell mass, metabolic activity and intracellular ATP levels compared to identical concentrations of MC_4_ (Figures S4A, S5A and S6A)_._ Similarly, a significantly superior reduction in cell mass occurred in the differentiated SH-SY5Y cells treated with MitoQ and SkQ_1_ compared to MC_4_ at concentrations higher than 1 and 3.2 μM, respectively (Figure S4C). In terms of metabolic activity, the cells treated with MitoQ and SkQ_1_ presented a superior reduction when compared to MC_4_ at concentrations higher than 3.2 and 6.3 μM (Figure S5C). Additionally, the cells treated with MitoQ and SkQ_1_ at 1 μM showed lower ATP values compared to cells treated with identical concentrations of MC_4_ (Figure S6C). Our results suggest that MC_4_ is less toxic than MitoQ and SkQ_1_ in HepG2 and SH-SY5Y cells.

Additionally, cells treated with concentrations above 1 μM of MitoQ and SkQ_1_ also showed a superior reduction in cell mass, metabolic activity and intracellular ATP levels compared with identical concentrations of MC_7.2_ (Figures S4A, S5A and S6A). However, the differentiated SH-SY5Y cells treated with MitoQ and SkQ_1_ presented a superior reduction in their mass, compared to cells treated with MC_7.2,_ at concentrations above 3.2 and 6.3 μM, respectively (Figure S4C). We found that the cells treated with MitoQ and SkQ_1_ also presented a superior reduction in metabolic activity compared to cells treated with MC_7.2_ at concentrations above 6.3 μM (Figure S5C). Likewise, the cells treated with MitoQ and SkQ_1_ at 1 μM showed lower ATP values compared to cells treated with identical concentrations of MC_7.2_ (Figure S6C). Together, these results suggest that MC_7.2_ presented lower cytotoxicity than mitochondria-targeted antioxidants MitoQ, SkQ_1_ in HepG2 and differentiated SH-SY5Y cells.

Analyzing the cytotoxic effects of non-targeted resveratrol or CoQ_10_ in the HepG2 cells, our data showed that resveratrol increased the cell mass at concentrations of 1 and 3.25 μM, and decreased it at concentrations above 25 μM compared to untreated cells (Figure 2B). On the other hand, CoQ_10_ did not affect the cell mass up to 100 μM (Figure 2B). Our results also showed that resveratrol reduced HepG2 metabolic activity above 50 μM. The HepG2 cells treated with CoQ_10_ showed increased metabolic activity at 6.3 and 50 μM compared to untreated cells (Figure 3B). On the other hand, the HepG2 cells treated with CoQ_10_ (25 and 50 μM) and resveratrol (25 μM) presented higher ATP levels (Figure 4B).

The loss of HepG2 cell mass that occurred with concentrations above 12.5 and 25 μM MC_3_ was comparable to the effect of identical concentrations of CoQ_10_ and resveratrol, respectively (Figure S4A). The cells treated with MC_3_ above 25 and 50 μM showed lower metabolic activity compared to similar concentrations of CoQ_10_ and resveratrol, respectively (Figure S5A). Additionally, cells treated with MC_3_ above 6.3 and 3.2 μM showed lower intracellular ATP levels than with similar concentrations of CoQ_10_ and resveratrol (Figure S6A).

In differentiated SH-SY5Y cells, our results showed that resveratrol decreased cell mass at concentrations above 25 μM compared to untreated cells, while CoQ_10_ did not affect cell mass up to 100 μM (Figure 2D). Resveratrol and CoQ_10_ did not affect the metabolic activity relative to untreated cells (Figure 3D). On the other hand, CoQ_10_ increased intracellular ATP levels in differentiated SH-SY5Y cells at concentrations of 1 and 3.2 μM. The differentiated SH-SY5Y cells treated with resveratrol showed increased intracellular ATP levels at 3.2 μM, and decreased levels at concentrations above 25 μM compared to untreated cells (Figure 4D). Compared with the non-targeted antioxidants, MC_3_ 100 μM resulted in a superior loss of cell mass to 100 μM CoQ_10_ (Figure S4C), without any difference in cell mass alterations between MC_3_ and resveratrol. No differences between MC_3_, CoQ_10_, or resveratrol were observed regarding metabolic activity (Figure S5C). The cells treated with MC_3_ 100 μM presented lower intracellular ATP levels compared to cells treated with 100 μM CoQ_10_, while no alterations in intracellular ATP levels were observed when comparing cells treated with resveratrol and MC_3_ (Figure S6C).

Regarding MC_6.2_, our results showed a superior loss of cell mass above 6.3 and 12.5 μM compared to similar concentrations of CoQ_10_ and resveratrol, respectively (Figure S4A,C). As observed in cell mass assays, cells treated with concentrations above 12.5 μM MC_6.2_ showed reduced metabolic activity and intracellular ATP levels compared to identical concentrations of CoQ_10_ and resveratrol (Figures S5A,C and S6A,C).

Additionally, no differences in cell mass and metabolic activity between CoQ_10_ and MC_4_ at concentrations up to 100 μM were found. The cells treated with resveratrol at concentrations above 25 μM presented a superior reduction in cell mass and metabolic activity compared to cells treated with identical concentrations of MC_4_ (Figures S4A and S5A). MC_4_ at concentrations up to 100 μM did not differ from the same concentrations of CoQ_10_ and resveratrol regarding its effects on HepG2 ATP levels (Figure S6A).

No differences in the effects of MC_4_ and CoQ_10_ at concentrations up to 100 μM on differentiated SH-SY5Y cell mass were observed. However, the cells treated with resveratrol at concentrations above 50 μM presented a superior reduction in cell mass compared to MC_4_ (Figure S4C). No differences in metabolic activity were observed when comparing the cells treated with MC_4_ and CoQ_10_ and resveratrol at concentrations up to 100 μM (Figure S5C). Additionally, cells treated with MC_4_ presented lower intracellular ATP levels when compared to CoQ_10_ at 100 μM, while cells treated with resveratrol at concentrations above 50 μM presented significantly lower intracellular ATP levels compared with identical concentrations of MC_4_ (Figure S6C). Our results suggest that MC_4_ is less toxic than resveratrol in HepG2 and SH-SY5Y cells.

Similarly, by comparing the cytotoxic effect of MC_7.2_ with that of non-targeted antioxidants, our results showed that MC_7.2_ at 100 μM led to a superior decrease in HepG2 cell mass compared to identical concentrations of CoQ_10_ (Figure S4A). By contrast, no differences in cell mass effects were observed when comparing cells treated with resveratrol and MC_7.2_ at concentrations up to 100 μM (Figure S4A). Likewise, no alterations were observed in metabolic activity between cells treated with MC_7.2_ and CoQ_10_ or resveratrol at concentrations up to 100 μM (Figure S5A). The cells treated with MC_7.2_ at 12.5 and 25 μM presented lower intracellular ATP levels compared to cells treated with identical concentrations of CoQ_10_ and resveratrol, respectively (Figure S6A). No differences were observed in differentiated SH-SY5Y cell mass and metabolic activity after treatment with CoQ_10_ and resveratrol up to 100 μM. Additionally, MC_7.2_ resulted in a superior decrease in intracellular ATP levels at 100 μM compared to cells treated with CoQ_10_ (Figure S6C). Additionally, the differentiated SH-SY5Y cells treated with MC_7.2_ at concentrations above 12.5 μM presented lower ATP levels compared to cells treated with identical concentrations of resveratrol (Figure S6C). These data suggest that MC_7.2_ presented higher cytotoxicity than CoQ_10_ and resveratrol in these two cell lines.

### 3.3. Comparative Effects of MitoCINs and Targeted- and Non-Targeted Antioxidants on Mitochondrial Membrane Potential and Cellular OCR/ECAR

Based on the previous results obtained with the four MitoCINs and targeted- and non-targeted antioxidants, we selected the two highest concentrations that showed no cytotoxicity or a reduction in cytotoxicity of less than 40% compared to untreated cells. The concentrations selected were: MC_3_: 12.5 and 25 μM for HepG2 and 25 and 50 μM for differentiated SH-SY5Y; MC_6.2_: 6.3 and 12.5 μM for HepG2 and 12.5 and 25 μM for differentiated SH-SY5Y; MC_4_: 50 and 100 μM for HepG2 and differentiated SH-SY5Y; MC_7.2_: 50 and 100 μM for HepG2 and 25 and 50 μM for differentiated SH-S cells; SkQ_1_: 0.5 and 1 μM for HepG2 and 1.5 and 3.1 μM for differentiated SH-SY5Y cells; CoQ_10_: 50 and 100 μM for HepG2 and differentiated SH-SY5Y cells; resveratrol: 25 and 50 μM for HepG2 and differentiated SH-SY5Y cells. The untreated cells, as well as non-cytotoxic concentrations of the parental or alkylTPP compounds, were also used as controls. Although the concentrations were different in several cases, our objective was to compare the mitochondrial effects of MitoCINs with the quinone-based mitochondria-targeted antioxidants and non-targeted antioxidants, at the highest non-toxic concentrations.

#### 3.3.1. Alterations in Mitochondrial Membrane Potential

The comparison between the mitochondrial membrane potentials of the HepG2 and differentiated SH-SY5Y cells treated with the three classes of compounds was determined indirectly by measuring cellular TMRM fluorescence intensity.

**Figure 5:**
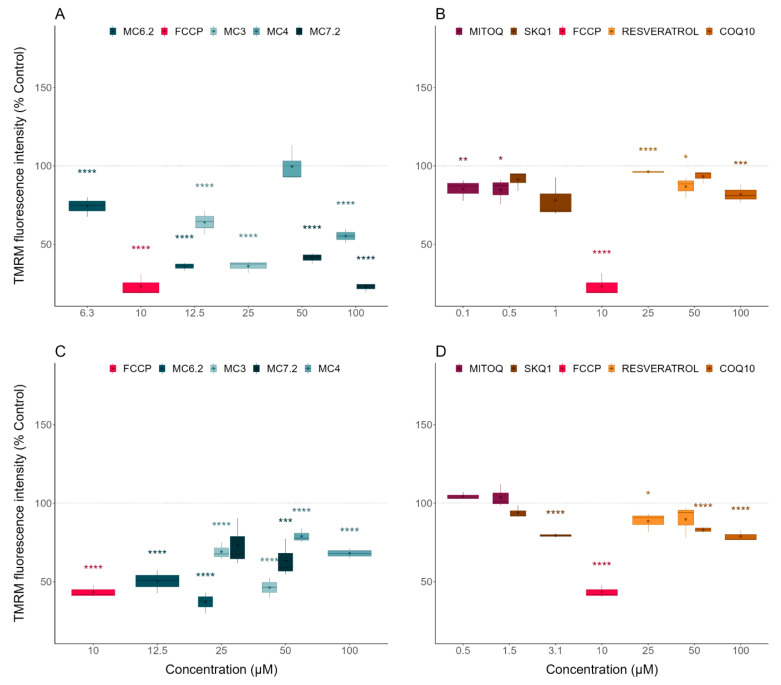
Effect of MitoCINs, quinone-based mitochondria-targeted and non-targeted antioxidants on TMRM intensity fluorescence. Human Caucasian hepatocyte carcinoma (HepG2, (**A**,**B**)) and differentiated human neuroblastoma (SH-SY5Y, (**C**,**D**)) were treated with increasing concentrations of the different molecules for a period of 48 h and mitochondrial membrane potential was indirectly evaluated using TMRM fluorescence intensity. Data are the mean± SE of three independent experiments and the results are expressed as percentage of the control (control = 100%). Statistically significant differences between control (CTL) and treated groups were evaluated using a *t*-test. **** *p* < 0.0001, *** *p* < 0.001, ** *p* < 0.01 and * *p* < 0.05 compared to the respective control (CTL).

The selected concentrations of MC_3_ (up to 40% reduction in HepG2 cells, up to 20% reduction in differentiated SH-SY5Y cells), MC_6.2_ (up to 40% reduction in HepG2 cells, up to 25% reduction in differentiated SH-SY5Y cells), MC_4_ (no effect on HepG2, up to 20% reduction in differentiated SH-SY5Y cells) and MC_7.2_ (up to 40% reduction in HepG2 cells, up to 20% reduction in differentiated SH-SY5Y cells) significantly reduced the mitochondrial membrane potential compared to untreated HepG2 (Figure 5A) or the differentiated SH-SY5Y cells (Figure 5C). The next step was to compare the compounds of the MitoCIN library with the two other classes of compounds. Comparing MC_3_ with the other mitochondria-targeted antioxidants, our results showed that the non-cytotoxic concentrations of MitoQ (0.1 and 0.5 µM) significantly decreased the mitochondrial membrane potential in the HepG2 cells (Figure 5B), albeit by a lower degree compared to MC_3_ (25 µM, Figure S7A). SkQ_1_ (0.5 and 1 µM, Figure 5B) had no effect on HepG2 mitochondrial membrane potential. In differentiated SH-SY5Y cells, SkQ_1_ (1.5 and 3.1 µM) significantly decreased the mitochondrial membrane potential compared to untreated cells (Figure 5D), again in a lower magnitude than MC_3_ (25 and 50 µM, Figure S7D). Comparing the extent of the effects of MC_6.2_ (12.5 μM for HepG2 and 12.5 and 25 μM for differentiated SH-SY5Y cells) with the other mitochondrial-targeted antioxidants, our results showed that SkQ_1_ (0.5 and 1 µM for HepG2 and 1.5 and 3.1 µM for differentiated SH-SY5Y cells) significantly decreased mitochondrial membrane potential in HepG2 (Figure S7A) and differentiated SH-SY5Y cells to a lesser extent than MC_6.2_ (Figure S7D). We also observed that MitoQ (0.1 and 0.5 µM) reduced mitochondrial membrane potential in HepG2 cells to a lesser extent than 12.5 μM MC_6.2_ (Figure S7A). Similarly, in differentiated SH-SY5Y cells, MC_6.2_ (12.5 and 25 μM) significantly decreased mitochondrial membrane potential to a greater extent than MitoQ (0.5 and 1.5 µM, Figure S7D).

Comparing MC_4_ with the quinone-based mitochondria-targeted antioxidants, we observed that 100 µM MC_4_ caused a significantly greater loss in mitochondrial membrane potential compared to MitoQ (0.1 and 0.5 µM) in HepG2 (Figure S7A) or to SkQ1 (1.5 and 3.1 µM) in the SH-SY5Y cells (Figure S7D). The HepG2 cells treated with 50 and 100 μM MC_7.2_ showed a significantly greater decrease in mitochondrial membrane potential compared to cells treated with MitoQ (0.1 and 0.5 μM) and SkQ_1_ (0.5 and 1 μM) (Figure S7A). The differentiated SH-SY5Y cells treated with 50 μM MC_7.2_ presented lower mitochondrial membrane potential compared to the cells treated with MitoQ (0.5 and 1.5 μM), but without significant alterations compared to the cells treated with SkQ_1_ (0.5 and 1 μM) (Figure S7D).

Comparing MC_3_ with the non-targeted antioxidants, our results showed that the non-cytotoxic concentrations of resveratrol (25 and 50 µM), CoQ_10_ (50 and 100 µM) significantly decreased mitochondrial membrane potential in HepG2 cells compared to untreated cells (Figure 5D), albeit with a lower magnitude when compared to MC_3_ (25 µM) (Figure S7A).

In the differentiated SH-SY5Y cells, resveratrol (25 and 50 µM) and CoQ_10_ (50 and 100 µM) also significantly decreased mitochondrial membrane potential compared to untreated cells (Figure 5D), with a lower amplitude than MC_3_ (50 µM) (Figure S7D).

Comparing the extension of effects of MC_6.2_ (12.5 μM for HepG2 and 12.5 and 25 μM for differentiated SH-SY5Y cells) with those of the non-targeted antioxidants, our results showed that CoQ_10_ (50 and 100 µM) and resveratrol (25 and 50 µM) significantly decreased mitochondrial membrane potential in HepG2 and differentiated SH-SY5Y cells, to a lesser extent than MC_6.2_ (Figure S7A,D).

Additionally, we observed that 100 µM MC_4_ caused a superior loss of mitochondrial membrane than resveratrol (25 and 50 µM) and CoQ_10_ (50 and 100 µM) in the HepG2 cells (Figure S7A) or when compared to resveratrol (25 µM) in the SH-SY5Y cells (Figure S7D).

The HepG2 cells treated with 50 and 100 μM MC_7.2_ presented reduced mitochondrial membrane potential compared to the cells treated with CoQ_10_ (50 and 100 μM) and resveratrol (25 and 50 μM) (Figure S7A). No alterations were observed in the differentiated SH-SY5Y cells treated with 50 and 100 μM MC_7.2_, compared to the cells treated with CoQ_10_ (50 and 100 μM) and resveratrol (25 and 50 μM) (Figure S7D), since these molecules decreased the mitochondrial membrane potential per se. Overall, the four MitoCINs significantly decreased the mitochondrial membrane potential to a greater extent than CoQ_10_, resveratrol, MitoQ and SkQ_1_.

The mitochondrial membrane potential of HepG2 and differentiated SH-SY5Y cells treated with MitoCINs parental compounds and their alkylTPP constituents were also measured to gain insights into cytotoxicity mechanisms. No alterations in mitochondrial membrane potential were observed in HepG2 cells treated with caffeic acid (50 μM), hydrocaffeic acid (100 μM), TriOH (50 and 100 μM) and hydro-TriOH (50 and 100 μM). However, the HepG2 cells treated with 50 μM hydrocaffeic acid showed increased mitochondrial membrane potential, while 100 μM caffeic acid reduced this parameter in the HepG2 cells (Figure S8A). In differentiated SH-SY5Y cells, caffeic acid (50 and 100 μM), TriOH (50 and 100 μM) and hydro-TriOH (50 μM) did not have any effect on mitochondrial membrane potential. As in the assays on HepG2 cells, differentiated SH-SY5Y cells treated with 50 μM hydrocaffeic acid showed an increase in mitochondrial membrane potential. On the other hand, differentiated SH-SY5Y cells treated with 100 μM hydrocaffeic acid or hydroTriOH presented reduced mitochondrial membrane potential compared to untreated cells (Figure S8C). In sum, our results showed that MC_3_, MC_6.2_ and MC_4_ reduced mitochondrial membrane potential compared to their parental compounds in both cell lines (Figure S7B,E). MC_7.2_ also reduced mitochondrial membrane potential compared to hydrocaffeic acid in HepG2 cells, without showing any alterations in the differentiated SH-SY5Y cells (Figure S7B,E).

Considering the alkylTPP compounds, no alterations in mitochondrial membrane potential were observed in HepG2 (Figure S4B) and differentiated SH-SY5Y cells (Figure S4D) treated with TPP-C6 (0.5 and 1 μM for HepG2 and 3.1 and 6.25 μM for differentiated SH-SY5Y cells), TPP-C8 (0.5 and 1 μM for HepG2 and 3.1 and 6.25 μM for differentiated SH-SY5Y cells) and TPP-C10 (0.1 μM for HepG2 and 1.5 μM for differentiated SH-SY5Y cells) compared to untreated cells. However, HepG2 cells treated with 0.01 μM TPP-C10 presented reduced mitochondrial membrane potential compared to untreated cells (Figure S4B), while the differentiated SH-SY5Y cells treated with 0.5 μM TPP-C10 showed an increase in mitochondrial membrane potential compared to untreated cells (Figure S4D). Comparing MitoCINs with their respective TPP cations, MC_3_, MC_6.2_, and MC_7.2_ reduced mitochondrial membrane potential in both cell lines (Figure S7C,F). However, MC_4_ only reduced mitochondrial membrane potential compared to TPP-C6 in the HepG2 cells (Figure S7C).

In sum, the MitoCINs significantly reduced mitochondrial membrane potential compared to their respective parental compounds and TPP cations in the HepG2 and differentiated SH-SY5Y cells (except for differentiated SH-SY5Y cells treated with MC_4_). They also reduced mitochondrial membrane potential to a greater extent than the non-targeted and quinone-based mitochondrial-targeted antioxidants at the highest non-cytotoxic concentrations. Table 1 shows a summary of the mitochondrial membrane potential results.

#### 3.3.2. Alterations in Cellular OCR and ECAR

The evaluation of the cytotoxicity of the different MitoCINs and the non-targeted and quinone-based mitochondria-targeted antioxidants on the OCR and ECAR of the HepG2 and differentiated SH-SY5Y cells was performed by using an Agilent-Seahorse XFe96 analyzer.

**Figure 6:**
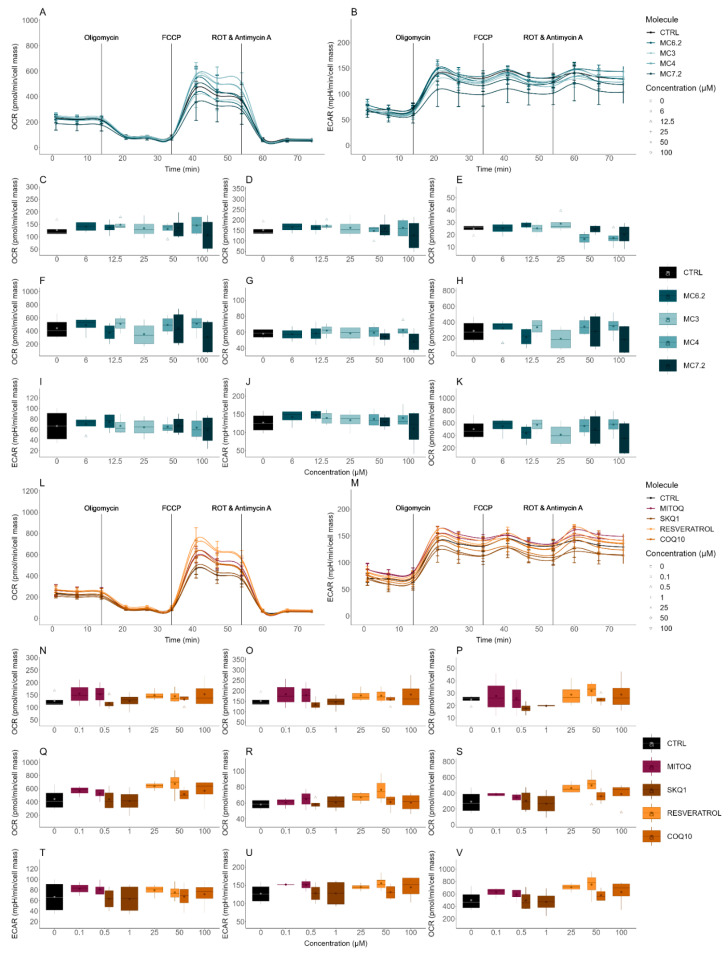
Effect of MitoCINs, quinone-based mitochondria-targeted and non-targeted antioxidants on oxygen consumption rate (OCR) and extracellular acidification rate (ECAR). OCR- and ECAR-associated parameters were assessed with a Seahorse XFe96 Extracellular Flux Analyzer. OCR (**A**,**L**) and ECAR (**B**,**M**) were assessed in human Caucasian hepatocyte carcinoma (HepG2) cells treated with increasing concentrations of the different molecules for a period of 48 h. Several OCR parameters were evaluated: ATP production-linked OCR (**C**,**N**), basal respiration (**D**,**O**), proton leak-based OCR (**E**,**P**), maximal respiration (**F**,**Q**), non-mitochondrial respiration (**G**,**R**) and spare respiratory capacity (**H**,**S**). ECAR parameters were also evaluated, including basal ECAR (**I**,**T**), stressed ECAR (**J**,**U**) and stressed OCR (**K**,**V**). Data are the mean± SE of four independent experiments and the results are expressed in the interquartile range (Q1–Q3) together with the (−) median. Statistically significant differences between control (CTL) and treated groups were evaluated using a *t*-test. Significance was accepted with *p* < 0.05.

**Figure 7:**
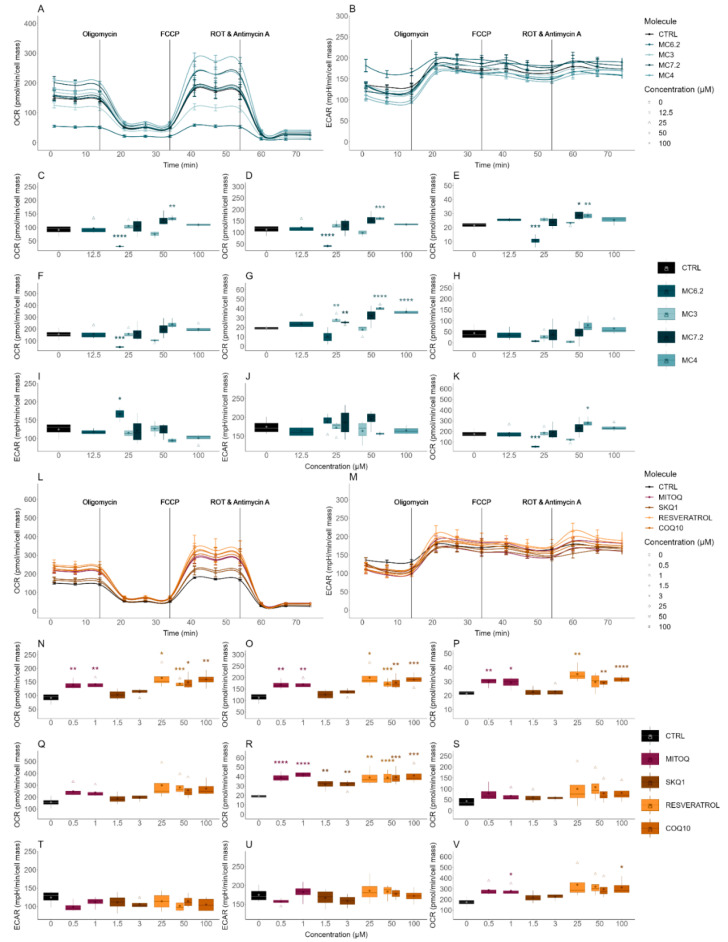
Effect of MitoCINs, quinone-based mitochondria-targeted and non-targeted antioxidants on oxygen consumption rate (OCR) and extracellular acidification rate (ECAR). OCR- and ECAR-associated parameters were assessed with a Seahorse XFe96 Extracellular Flux Analyzer. OCR (**A**,**L**) and ECAR (**B**,**M**) were assessed in differentiated human neuroblastoma (SH-SY5Y) cells treated with increasing concentrations of the different molecules for a period of 48 h. Several OCR parameters were evaluated: ATP production-linked OCR (**C**,**N**), basal respiration (**D**,**O**), proton leak-based OCR (**E**,**P**), maximal respiration (**F**,**Q**), non-mitochondrial respiration (**G**,**R**) and spare respiratory capacity (**H**,**S**). ECAR parameters were also evaluated, including basal ECAR (**I**,**T**), stressed ECAR (**J**,**U**) and stressed OCR (**K**,**V**). Data are the mean ± SE of four independent experiments and the results are expressed in the interquartile range (Q1–Q3) together with the (−) median. Statistically significant differences between control (CTL) and treated groups were evaluated using a *t*-test. **** *p* < 0.0001, *** *p* < 0.001, ** *p* < 0.01 and * *p* < 0.05 compared to the respective control (CTL).

MC_3_ (12.5 and 25 µM), MC_6.2_ (6.3 and 12.5 µM), MC_4_ (50 and 100 µM) and MC_7.2_ (50 and 100 µM) did not induce any alterations in the OCR and ECAR parameters in the HepG2 cells (Figure 6A–K). Similarly, no differences were found in the OCR and ECAR parameters in the differentiated SH-SY5Y cells treated with MC_3_ (25 and 50 µM) compared to untreated cells (Figure 7A–K). We observed that in these cells, 25 µM MC_6.2_ reduced the ATP production-linked OCR, proton leak-associated OCR, basal, maximal, and stressed OCR and increased the basal ECAR (Figure 7A–K). On the other hand, 50 µM of MC_4_ increased the ATP production-linked OCR, proton leak-associated OCR, non-mitochondrial respiration, basal and stressed OCR in the differentiated SH-SY5Y cells. MC_4_ (100 µM) also increased non-mitochondrial respiration (Figure 7A–K). For MC_7.2_ (50 µM) our results showed an increase in proton leak and non-mitochondrial respiration (Figure 7A–K).

By analyzing the OCR and ECAR parameters of the two mitochondria-targeted antioxidants in clinical trials, our results showed that HepG2 cells treated with MitoQ (0.5 and 1 µM) and SkQ_1_ (0.5 and 3 µM) did not show any alterations in their endpoints compared to untreated cells (Figure 6L–V) or with cells treated with MC_3_ (12.5 and 25 µM) (Figure S9A–K).

When comparing the effects on differentiated SH-SY5Y cells, we observed that MC_3_ 50 µM presented lower values of ATP production-linked OCR, proton leak-associated OCR, basal, non-mitochondrial, maximal and stressed OCR compared to the cells treated with MitoQ (0.5 and 1 µM) (Figure S10A–K). The cells treated with 50 µM MC_3_ also presented lower spare respiratory capacity than the cells treated with MitoQ (0.5 and 1 µM) (Figure S9H). The cells treated with 1.5 µM SkQ_1_ presented higher spare respiratory capacity than cells treated with 50 µM MC_3_, while cells treated with 3 µM SkQ_1_ presented higher spare respiratory capacity and maximal and stressed OCR, compared to the cells treated with 50 µM MC_3_ (Figure S10A–K).

No differences were observed in OCR and ECAR between HepG2 cells treated with MC_6.2_ (12.5 and 25 µM) and MitoQ (0.5 and 1 µM) (Figure S9A–K). However, cells treated with 1 µM SkQ_1_ presented lower proton leak-associated OCR values than the HepG2 cells treated with 12.5 µM MC_6.2_ (Figure S9E). On the other hand, the differentiated SH-SY5Y cells treated with 25 µM MC_6.2_ presented lower ATP production-linked OCR, proton leak-associated OCR, basal, maximal, non-mitochondrial and stressed OCR compared to the cells treated with MitoQ (0.5 and 1 µM) and SkQ_1_ (1.5 and 3 µM) (Figure S10A–K). The cells treated with MitoQ (0.5 and 1 µM) and 3 µM SkQ_1_ showed lower basal ECAR values than cells treated with 25 µM MC_6.2_ (Figure S10I). The spare respiratory capacity was also decreased in differentiated SH-SY5Y cells treated with 25 µM MC_6.2_ compared to cells treated with MitoQ (0.5 and 1 µM) and SkQ_1_ (1.5 and 3 µM) (Figure S10H). Comparing MC_4_ with mitochondria-targeted antioxidants, no differences were observed when comparing HepG2 cells treated with MC_4_ (50 and 100 μM), MitoQ (0.1 and 0.5 μM for HepG2 and 0.5 and 1 µM for differentiated SH-SY5Y cells) and SkQ_1_ (0.5 and 1 µM for HepG2 and 1.5 and 3 µM for differentiated SH-SY5Y cells) (Figures S9 and S10). On the other hand, HepG2 cells treated with MC_7.2_ (50 and 100 µM) presented similar values of OCR and ECAR compared to cells treated with MitoQ (0.1 and 0.5 µM) and 0.5 µM SkQ_1_ (Figure S9A–K). The cells treated with 25 µM MC_7.2_ also presented a significant reduction in non-mitochondrial respiration compared to cells treated with 0.5 and 1 µM MitoQ (Figure S10G). In sum, MC_3_ and MC_6.2_ lowered ATP production-linked OCR, proton leak-associated OCR, basal, maximal and stressed OCR compared with MitoQ and SkQ1, while no alterations in any parameter were observed in HepG2 and differentiated SH-SY5Y cells treated with MC_4_ and MC_7.2_ or MitoQ and SkQ_1_.

The HepG2 cells treated with resveratrol (25 and 50 µM) and CoQ_10_ (50 and 100 µM) did not show any differences in OCR and ECAR parameters compared to untreated cells (Figure 6L–V) or with the cells treated with MC_3_ (12.5 and 25 µM) (Figure S9L–V). The differentiated SH-SY5Y cells treated with 50 µM MC_3_ presented lower values of ATP production-linked OCR, proton leak-associated OCR, basal, non-mitochondrial, maximal and stressed OCR compared to the cells treated with resveratrol (25 and 50 µM) and CoQ_10_ (50 and 100 µM) (Figure S10L–V).

In HepG2 cells, no differences were observed between MC_6.2_ (12.5 and 25 µM), resveratrol (25 and 50 µM) and CoQ_10_ (50 and 100 µM). The differentiated SH-SY5Y cells treated with 25 µM MC_6.2_ presented reduced ATP production-linked OCR, proton leak-associated OCR, basal, maximal, non-mitochondrial and stressed OCR compared to the cells treated with resveratrol (25 and 50 µM) and CoQ_10_ (50 and 100 µM) (Figure S10L–V). Interestingly, differentiated SH-SY5Y cells treated with 25 µM MC_6.2_ also showed higher basal ECAR values compared to those treated with resveratrol (50 µM) and CoQ_10_ (50 and 100 µM) (Figure S10T).

No differences were observed in OCR and ECAR when comparing HepG2 cells treated with MC_4_ (50 and 100 μM), resveratrol (25 and 50 µM) or CoQ_10_ (50 and 100 μM) (Figure S9L–V). However, differentiated SH-SY5Y cells treated with 100 μM MC_4_ presented lower basal respiration compared to cells treated with 50 μM resveratrol and 100 μM CoQ_10_ (Figure S10O).

The HepG2 cells treated with MC_7.2_ (50 and 100 µM) presented similar OCR and ECAR values compared to the cells treated with resveratrol (25 and 50 µM) and CoQ_10_ (50 and 100) (Figure S9L–V). The differentiated SH-SY5Y cells treated with 25 µM MC_7.2_ also presented a significant reduction in non-mitochondrial respiration compared to the cells treated with 50 µM resveratrol (Figure S10R). Our results suggest that HepG2 cells treated with these four MitoCINs presented similar OCR and ECAR values compared to the cells treated with resveratrol and CoQ_10_. In differentiated SH-SY5Y cells, MC_3_ and MC_6.2_ reduced ATP production-linked OCR, proton leak-associated OCR, basal, maximal and stressed OCR compared to resveratrol and CoQ_10_.

The OCR and ECAR parameters of HepG2 and differentiated SH-SY5Y cells treated with parental compounds of all four MitoCINs and their alkylTPP constituents were also measured to gain insight into the cytotoxicity mechanisms of the MitoCIN compounds.

No differences in OCR and ECAR parameters were observed in HepG2 cells treated with caffeic acid (50 and 100 µM), hydrocaffeic acid (50 and 100 μM), TriOH (50 µM) and hydro.Tri-OH (50 and 100 µM) (Figure S11A–K). However, 100 μM TriOH decreased proton leak-associated OCR in HepG2 cells compared to untreated cells (Figure S11A–K). In differentiated SH-SY5Y cells, 50 µM caffeic acid increased the ATP production-linked OCR, proton leak-associated OCR, basal, non-mitochondrial, maximal and stressed OCR, while 100 µM caffeic acid increased the proton leak-associated OCR and non-mitochondrial OCR (Figure S12A–K). Our results also showed that hydrocaffeic acid (50 and 100 μM) increased the ATP production-linked, proton leak-associated OCR, basal, maximal, non-mitochondrial and stressed OCR compared to untreated cells (Figure S12A–K). TriOH (25 and 50 µM) increased the non-mitochondrial respiration compared to untreated cells (Figure S12A–K). The differentiated SH-SY5Y cells treated with 50 µM hydro-TriOH increased the ATP production-linked OCR and proton leak-associated OCR compared to untreated cells. In comparison, 100 µM hydro-TriOH increased the ATP production-linked OCR, basal, proton leak-associated OCR, non-mitochondrial respiration, maximal and stressed OCR compared to untreated cells, without affecting the spare respiratory capacity (Figure S12A–K). No differences in these parameters were observed between HepG2 cells treated with MC_3_ (12.5 and 25 µM), MC_6.2_ (6.3 and 12.5 µM), MC_4_ (50 and 100 µM) and MC_7.2_ (50 and 100 µM) compared to their respective parental compounds (Figure S13A–K). In differentiated SH-SY5Y cells, MC_3_ (25 and 50 µM) and MC_6.2_ (25 µM) presented lower ATP production-linked OCR, proton leak-associated OCR, basal, non-mitochondrial and stressed OCR compared to cells treated with their respective parental compounds (Figure S14A–K), while MC_4_ (50 and 100 µM) and MC_7.2_ (25 and 50 µM) presented similar OCR and ECAR parameters relative to their parental compounds (Figure S14L–V).

Considering alkylTPP compounds, no differences were measured between OCR and ECAR parameters in HepG2 cells treated with TPP-C6 (0.5 and 1 µM), TPP-C8 (0.5 and 1 µM) and TPP-C10 (0.01 and 0.1 µM) compared to untreated cells (Figure S11L–M). The differentiated SH-SY5Y cells treated with 3 µM TPP-C6 presented a significant reduction in proton leak-associated OCR, while 6 µM TPP-C6 reduced ATP production-linked, proton leak-associated OCR, basal, maximal, non-mitochondrial and stressed OCR and also increased the basal ECAR compared to untreated cells (Figure S12L–M). The differentiated SH-SY5Y cells treated with 6 µM TPP-C8 presented reduced ATP production-linked OCR, proton leak-associated OCR, basal, non-mitochondrial, maximal and stressed OCR compared to untreated cells, as well as an increase in basal ECAR compared with the untreated cells (Figure S12L–M). On the other hand, 1 µM TPP-C10 also decreased the ATP production-linked OCR, proton leak-associated OCR, basal, maximal, and stressed OCR compared to untreated cells (Figure S12L–M). In HepG2 cells, no differences in OCR and ECAR parameters were found between the cells treated with MC_3_ (12.5 and 25 µM), MC_6.2_ (6.3 and 12.5 µM), MC_4_ (50 and 100 µM) and MC_7.2_ (50 and 100 µM) and their respective alkylTPP cations (Figure S15A–K). Interestingly, the differentiated SH-SY5Y cells treated with MC_3_ (25 and 50 µM), MC_6.2_ (25 µM), MC_4_ (50 and 100 μM) and MC_7.2_ (50 µM) presented higher ATP production-linked OCR, proton leak-associated OCR, basal OCR compared to the cells treated with their respective TPP cations (Figure S16A–K).

In sum, MC_3_ and MC_6.2_ affected mitochondrial function in the HepG2 and differentiated SH-SY5Y cells more significantly than the non-targeted and quinone-based mitochondrial-targeted antioxidants (MC_3_: 12.5–50 μM and MC_6.2_: 6.3–25 μM vs. SkQ1: 0.5–3.1 μM and MitoQ: 0.5–1 µM), while MC_4_ and MC_7.2_ presented similar effects on mitochondrial function in both cell lines compared to the non-targeted and mitochondrial-targeted antioxidants (MC_4_: 50–100 μM and MC_7.2_: 25–100 μM vs. SkQ1: 0.5–3.1 μM, MitoQ: 0.5–1 µM, resveratrol: 25–50 μM and CoQ_10_: 50–100 μM).

## 4. Discussion

The prevention of mitochondrial oxidative damage is a promising pharmacological strategy to delay the progression of oxidative stress-related diseases [1,35]. Based on this approach, targeting mitochondria with molecules functionalized with a TPP^+^ moiety is a useful strategy for targeting different antioxidants inside the organelle [1,50]. Despite the relevance of mitochondrial-targeting drugs, several cytotoxic effects have been reported for a variety of systems, mostly related to ROS overproduction, membrane disruption and mitochondrial depolarization [5].

The TPP^+^-based mitochondria-targeted antioxidants MitoQ and SkQ_1_, based on coenzyme Q and plastoquinone moieties, respectively, both covalently linked to TPP^+^-C10, are two of the most commonly studied mitochondria-targeted antioxidants. Despite positive results in preclinical stages, both molecules present toxicity concerns, which are mainly related to the presence in the structure of a prooxidant quinone core [51,52,53]. As an alternative to MitoQ and SkQ_1_, a library of antioxidants based on HCAs and analogs linked to alkyl TPP^+^ was developed [23,24,25]. By extending our previous studies of this type of compound, our objective was to evaluate the cytotoxicity and effects on mitochondrial function of selected TPP^+^-based antioxidants from the MitoCINs library (MC_3_, MC_6.2_, MC_4_ and MC_7.2_) and compare them with the outline obtained under the same experimental conditions for MitoQ and SkQ_1_ and with two non-targeted antioxidants that have been under clinical trials, resveratrol and CoQ_10_. We used HepG2 and differentiated SH-SY5Y cells as cell models for this study, since they are often used in preclinical safety assessment of drug candidates [41,42,43].

The four MitoCINs under study are structurally related and based on HCAs or their analog present in the diet. Briefly, the chemical modifications were performed on the phenolic ring substitution pattern, on the type of spacer between the carboxamide and the phenolic ring and on the length of the alkyl linker between the carboxamide and the TPP moiety (Figure 1). The selected compounds feature different aromatic ring substituent patterns (dihydroxy (catechol) or trihydroxy (pyrogallol) moieties), lengths of alkyl linker (6- (*n* = 1), 8- (*n* = 2) and 10-carbon linker (*n* = 3)) and type of the spacer between the carboxamide and the aromatic nucleus (ethylene (R’ = −H_2_C−CH_2_−) or vinyl group (R’ = −HC=CH−)). (Figure 1) The importance of the diverse structural motifs in MItoCIN cytotoxicity was explored in this work.

We initially evaluated the cytotoxic profile of the four MitoCINs by determining their effects on cell mass, metabolic activity, and intracellular ATP levels in HepG2 and differentiated SH-SY5Y cells. Our results showed that MC_3_ presented toxicity above 12.5 and 50 μM in the HepG2 and differentiated SH-SY5Y cells, respectively. MC_6.2_ showed about 2 times more cytotoxicity in the same cell lines, namely at 6.3 and 25 μM, respectively. MC4 was the least toxic molecule, not showing toxicity in HepG2 cells and only displaying toxicity in the differentiated SH-SY5Y cells above 100 μM. MC_7.2_ also showed low cytotoxicity at concentrations above 100 μM in both cell lines (Figure 2, Figure 3 and Figure 4).

In general, MitoCINs showed a similar or higher cytotoxicity than the parental compounds and lower cytotoxicity than the alkylTPP substructures. In fact, no cytotoxicity was observed in the following parental compounds in the HepG2 and differentiated SH-SY5Y cells: caffeic acid, hydrocaffeic acid and hydro.-TriOH. TriOH reduced cell viability when tested at concentrations higher than 25 μM in the differentiated SH-SY5Y cells. Regarding the alkylTPP molecules, the order of cytotoxicity was established as TPP-C6 < TPP-C8 < TPP-C10. This data suggests that the cytotoxicity of the MitoCINs under study may be associated with the lipophilicity of the spacer and/or the presence of a TPP moiety and has little, if any, association with the presence of catechol or pyrogallol moieties, as previously described [54]. It should be noted that the extension of TPP^+^ derivative anchorage to the mitochondrial internal membrane is dependent upon their overall lipophilicity, which is mainly modulated by the length of the linker unit and also by the functionalization of the bioactive molecule.

Importantly, we observed higher cytotoxicity from both MitoQ and SkQ_1_ compared to resveratrol and CoQ_10_, or to MitoCINs at concentrations above 1 μM, in the HepG2 and differentiated SH-SY5Y cells (Figure 2, Figure 3 and Figure 4). In agreement with this finding, Reddy et al. demonstrated that MitoQ concentrations above 0.3 µM are toxic to neuronal cells [55], indicating that optimizing MitoQ dosage is a critical step for balancing toxicity and efficacy [56]. Additionally, Sukhanova et al. also demonstrated that the accumulation of MitoQ and SkQ_1_ in isolated yeast mitochondria can lead to toxic events related to prooxidant effects, depending on the dose [57], reinforcing the importance of developing mitochondrial compounds with lower toxicity and higher efficacy.

A summary of the data obtained in this study was built using violin plots. Figure S17 shows a compilation of cell viability studies, aggregating cell mass, metabolic activity and ATP concentrations measured in HepG2 (panel A) and differentiated SH-SY5Y (panel B) cells after being treated with MitoCINs, quinone-based mitochondria-targeted and non-targeted antioxidants at different concentrations. Each data point corresponds to the average value for the cell viability endpoints measured after treatment. The figure shows that the cell responses to different treatments notably varied at higher concentrations (broader distributions after 25 μM for HepG2 and 50 μM for SH-SY5Y), reinforcing the compounds’ different toxicity profiles. Furthermore, this figure shows that experimental cytotoxicity endpoints appeared to be positively correlated i.e., under a given treatment, these measurements tended to cluster together.

By comparing data on the cytotoxicity of the MitoCINs in HepG2 and differentiated SH-SY5Y cells, based on decreased cell mass, metabolic activity and ATP, a toxicity ranking was established: MC_4_ < MC_7.2_ < MC_3_ < MC_6.2_, as observed individually for each assay in Table 1. Notably, MC_4_ not only did not show any cytotoxic effect between 1–100 μM in the HepG2 and differentiated SH-SY5Y cells, which was in line with our previous results [23,25], but also improved several of the cellular parameters analyzed in this work. After analyzing the relationship between structure and toxicity, we can propose that the lower alkyl chain (C6-alkyl chain) and pyrogallol aromatic pattern are relevant substructures with which to further develop safer mitochondriotropic antioxidants.

As previously described, the presence of the TPP cation and a lipophilic spacer is essential for efficient mitochondrial accumulation, but a suitable lipophilic balance must be obtained to reduce the toxicity of mitochondrial-targeted antioxidants [54]. Particularly, we found that in cinnamic compounds or analogs, the catechol vs. pyrogallol replacement in combination with the type of spacer and a suitable length of the alkyl chain can clearly modify their redox behavior towards mitochondrial components, significantly affecting their biological outcome. In fact, the catecholic compounds MC_3_ and MC_6.2_ could be oxidized in situ to quinone, a species that can intervene in redox cycles, leading to an increase in ROS intracellular generation and, consequently, an increase in harmful cellular effects [16]. The current work clarified the relevance of the pyrogallol group, namely in MC_4_ and MC_7.2_, to modulate the mentioned redox cycle circumventing the cytotoxic events inherent in this type of compound.

Considering the effect of selected MitoCINs on mitochondrial function, our results showed that non-cytotoxic concentrations of MC_3_, MC_6.2_, MC_4_ and MC_7.2_ promoted an apparent loss of ΔΨ in HepG2 and differentiated SH-SY5Y cells (Figure S8B), most likely as a consequence of their accumulation in the mitochondrial matrix, as previously described for lipophilic triphenylphosphonium cations [58] and other mitochondria-targeted compounds, namely MitoQ, MitoE and MitoTempol [59]. In fact, lipophilic TPP cationic derivatives are rapidly and extensively taken up in vivo by mitochondria in a process driven by the large negative-inside ΔΨm. Although the uptake mechanism is still not well understood, the extent of the accumulation of a TPP^+^ based derivative depends on the plasma ΔΨ and ΔΨm, the volume of the cell, the external media and the number of mitochondria within a given cell [59]. Consequently, the amount of compound accumulated in mitochondria is dependent on the cell type and, accordingly, can explain the different effects of the TPP-based compounds on HepG2 and differentiated SH-SY5Y cells. The drug-metabolic capacity of HepG2 [60,61] cannot be excluded as a factor contributing to the specific effects of all the compounds tested in this study. On the other hand, we found that non-cytotoxic concentrations of MC_3_, MC_6.2_, MC_4_ and MC_7.2_ reduced the mitochondrial membrane potential in the HepG2 and differentiated SH-SY5Y cells, with a higher magnitude of effects compared to non-cytotoxic concentrations of MitoQ, resveratrol or SkQ_1_, resveratrol, and CoQ_10_, respectively, probably as a consequence of a higher accumulation on the mitochondrial matrix compared to non-cytotoxic concentrations of the other TPP-based compounds and commercial antioxidants. Interestingly, by analyzing non-cytotoxic concentrations of catechol and pyrogallol derivatives, it was noticeable that the MitoCINs conjugated with TPP-C10 resulted in a greater decrease in mitochondrial membrane potential compared to compounds conjugated with a TPP-C8 and TPP-C6 linker in the differentiated SH-SY5Y cells. In fact, a decrease in mitochondrial membrane potential does not always mean cytotoxicity. Still, our data on intracellular ATP levels suggest that some of the compounds’ effects on membrane potential are related to a decrease in ATP levels as well. In sum, these results also suggest that the length of the TPP alkyl chain can influence the capacity of these compounds to be accumulated. In sum, these results also suggest that the length of the TPP alkyl chain can influence the capacity of these compounds to be accumulated and cause alterations in mitochondria.

Regarding the cellular OCR and ECAR parameters, we concluded that MC_3_, MC_6.2,_ MC_4_ and MC_7.2_ did not present significant mitochondrial toxicity at concentrations up to 100 μM in the HepG2 cells (Figure 6). By contrast, a decrease in mitochondrial respiration in the differentiated SH-SY5Y cells treated with MC_6.2_ was found, which was observed through the decrease in ATP production-linked, proton leak, basal, maximal and stressed OCR compared to the untreated cells (Figure 7A–K). The difference in effects between the two types of cell probably occurred due to the amount of compound accumulated inside the mitochondria [59]. The reduction in maximal and stressed OCR suggests an impairment of non-phosphorylative respiration. At the same time, the inhibition of production-linked ATP might indicate an effect in the mitochondrial phosphorylative system at concentrations lower than those that caused the effects on cell viability. It should be noted that the decrease in the proton leak and basal OCR and the increase in the basal ECAR observed in the differentiated SH-SY5Y cells treated with MC_6.2_ probably occurred as a consequence of membrane-related effects. The decrease in the mitochondrial membrane potential may have resulted from the accumulation of this compound in the mitochondria and as a consequence of interactions with non-phosphorylative respiration, either at the protein or membrane levels. MC_3_ did not affect mitochondrial bioenergetics in the differentiated SH-SY5Y cells at the concentrations used. However, at the same concentrations, MC_3_ presented higher values of proton leak-associated OCR and lower values of ECAR, compared to the maximal non-cytotoxic concentrations of the respective alkylTPP cation (TPP-C8).

Nonetheless, 50 µM of pyrogallol-based derivative MC_4_ improved mitochondrial bioenergetics in the differentiated SH-SY5Y (but not HepG2) cells compared to untreated cells, as was manifested by the significant increase in the ATP production-linked OCR (Figure 7C). In parallel, MC_4_ and MC_7.2_ also increased the proton leakage through the mitochondrial inner membrane (IMM) (Figure 7E) resulting from a membrane permeabilization effect or a proton shuttling activity, which may consequently have reduced the mitochondrial membrane potential in these cells (Figure 5). The increase in proton leak-based OCR (Figure 7E) was not only due to the effect of the TPP cations, since this parameter was significantly higher in the cells treated with MC_4_ and MC_7.2_ compared to the cells treated with their corresponding alkylTPP compound. These data suggest that the effects on mitochondrial function resulted from the balance of their overall lipophilicity, which is linked to the length of the linker unit, the type of spacer and the structure of the bioactive molecule.

MC_3_ and MC_6.2_ affected mitochondrial respiration more significantly than resveratrol or CoQ_10_ at equal or lower concentrations (MC_3_: 25–50 μM and MC_6.2_: 12.5–25 μM vs. CoQ_10_: 50–100 μM and resveratrol: 25–50 μM)_._ The decline in mitochondrial respiration was confirmed by the decrease in ATP production-linked OCR, proton leak-associated OCR, basal, maximal and stressed OCR values comparatively to resveratrol or CoQ_10_. MC_3_ and MC_6.2_ also affected mitochondrial respiration more significantly than MitoQ and SkQ_1_, albeit at concentrations 12 to 50 times higher than these two mitochondrial-targeted antioxidants (MC_3_: 25–50 μM and MC_6.2_: 12.5–25 μM vs. SkQ1: 1.5–3.1 μM and MitoQ: 0.5–1 µM).

Analyzing in detail the effects of MC_4_ and MC_7.2_ compared to MitoQ and SkQ_1_, it is clear that at maximal non-cytotoxic concentrations of these four mitochondrial-targeted antioxidants, no mitochondrial toxicity was observed in the HepG2 and differentiated SH-SY5Y cells, although MC_4_ and MC_7.2_ showed around 100 and 1000 times less cytotoxicity in these two cell lines than SkQ_1_ and MitoQ, respectively (MC_4_: 50–100 μM and MC_7.2_: 25–100 μM vs. SkQ1: 0.5–3.1 μM and MitoQ: 0.5–1 µM). Despite this, MC_4_ decreased basal respiration and proton leak-associated OCR in the differentiated SH-SY5Y cells compared to resveratrol and CoQ_10_ at concentrations two times higher or identical to these two antioxidants, respectively (MC_4_: 50–100 μM vs. resveratrol: 25–50 μM and CoQ_10_: 50–100 μM). These results suggest that, despite being a cationic molecule, MC_4_ induced less proton leakage through the IMM, compared to CoQ_10_ and resveratrol.

In conclusion, MC_4_ and MC_7.2_ presented up to eight times less cytotoxicity than MC_3_ and MC_6.2_ and around 100 to 1000 times less cytotoxicity than SkQ_1_ and MitoQ in HepG2 and differentiated SH-SY5Y cells. Maximal non-cytotoxic concentrations (50 or 100 μM) of MC_4_ and MC_7.2_ did not induce any mitochondrial toxicity in HepG2 or differentiated SH-SY5Y cells. Additionally, the maximal non-cytotoxic concentrations of SkQ_1_ and MitoQ were around 100 to 1000 times less than the corresponding concentration of MC_4_ and MC_7.2_. In sum, based on the cytotoxicity cellular data and mitochondrial functional parameters analyzed, MC_4_ and MC_7.2_ emerged as potential drug candidates with a potential therapeutic application in mitochondrial oxidative stress-related diseases, particularly in hepatic and neurodegenerative diseases, presenting a better safety profile than the two quinone-based mitochondria-targeted molecules used for comparison.

## Figures and Tables

**Table 1:** Comparison of cytotoxicity and mitochondrial effects of MitoCIN compounds in HepG2 and differentiated SH-SY5Y cells.

Assay	Key Finding	Potency/Effect Size
Sulforhodamine B	Cell mass/Cytotoxicity	HepG2: MC_4_ < MC_7.2_ < MC_3_ < MC_6.2_ SH-SY5Y: MC_4_ ≈ MC_7.2_ < MC_3_ < MC_6.2_
Resazurin	Cell metabolic activity/Cytotoxicity	HepG2: MC_4_ < MC_7.2_ < MC_3_ < MC_6.2_ SH-SY5Y: MC_4_ ≈ MC_7.2_ < MC_3_ < MC_6.2_
Intracellular ATP levels	Cytotoxicity	HepG2: MC_4_ = MC_7.2_ < MC_3_ ≈ MC_6.2_ SH-SY5Y: MC_4_ < MC_7.2_ ≈ MC_3_ < MC_6.2_
TMRM	Mitochondrial polarization	HepG2: MC_4_ < MC_6.2_ ≈ MC_3_ < MC_7.2_ SH-SY5Y: MC_4_ ≈ MC_7.2_ < MC_3_ < MC_6.2_
Cellular OCR/ECAR	Cellular respiration and indirect measurement of cellular glycolytic activity	HepG2: MC_4_ ≈ MC_7.2_ ≈ MC_3_ ≈ MC_6.2_ SH-SY5Y: MC_4_ < MC_7.2_ < MC_3_ < MC_6.2_

TMRM: tetramethylrhodamine methyl ester, OCR: oxygen consumption rate, ECAR: extracellular acidification rate.

## Data Availability

Data supporting the results are available upon reasonable requests.

## Supplementary Materials

The following are available online at https://www.mdpi.com/article/10.3390/biom11111605/s1, Figure S1: Effect of parental antioxidants and alkylTPP compounds on cell mass, Figure S2: Effect of parental antioxidants and alkylTPP compounds on metabolic activity, Figure S3: Effect of parental antioxidants and alkylTPP compounds on ATP intracellular concentrations, Figure S4: Comparison between the effects of MitoCINs, quinone-based mitochondria-targeted, non-targeted antioxidants, parental antioxidants and alkylTPP compounds on cell mass, Figure S5: Comparison between the effects of MitoCINs, quinone-based mitochondria-targeted, non-targeted antioxidants, parental antioxidants and alkylTPP compounds on metabolic activity, Figure S6: Comparison between the effects of MitoCINs, quinone-based mitochondria-targeted, non-targeted antioxidants, parental antioxidants and alkylTPP compounds on ATP intracellular concentrations, Figure S7: Comparison between the effects of MitoCINs, quinone-based mitochondria-targeted, non-targeted antioxidants, parental antioxidants and alkylTPP compounds on TMRM intensity fluorescence, Figure S8: Effect of parental antioxidants and alkylTPP compounds on TMRM intensity fluorescence, Figure S9: Comparison between the effects of MitoCINs, quinone-based mitochondria-targeted and non-targeted antioxidants on oxygen consumption rate (OCR) and extracellular acidification rate (ECAR) in human caucasian hepatocyte carcinoma (HepG2) cells, Figure S10: Comparison between the effects of MitoCINs, quinone-based mitochondria-targeted and non-targeted antioxidants on oxygen consumption rate (OCR) and extracellular acidification rate (ECAR) in differentiated human neuroblastoma (SH-SY5Y) cells, Figure S11: Effect of parental antioxidants and alkylTPP compounds on oxygen consumption rate (OCR) and extracellular acidification rate (ECAR), Figure S12: Effect of parental antioxidants and alkylTPP compounds on oxygen consumption rate (OCR) and extracellular acidification rate (ECAR), Figure S13: Comparison between the effects of MitoCINs and parental antioxidants on oxygen consumption rate (OCR) and extracellular acidification rate (ECAR) in human caucasian hepatocyte carcinoma (HepG2) cells, Figure S14: Comparison between the effects of MitoCINs and parental antioxidants on oxygen consumption rate (OCR) and extracellular acidification rate (ECAR) in differentiated human neuroblastoma (SH-SY5Y) cells, Figure S15: Comparison between the effects of MitoCINs and alkylTPP compounds on oxygen consumption rate (OCR) and extracellular acidification rate (ECAR) in human caucasian hepatocyte carcinoma (HepG2) cells, Figure S16: Comparison between the effects of MitoCINs and alkylTPP compounds on oxygen consumption rate (OCR) and extracellular acidification rate (ECAR)in differentiated human neuroblastoma (SH-SY5Y) cells, Figure S17: Violin plot distributions of the average experimental points for HepG2 (panel A) and SH-SY5Y (panel B) viability, namey, cell mass (SRB), metabolic activity (resazurin) and ATP, under different compound treatments (MitoCINs, quinone-based mitochondria-targeted and non-targeted antioxidants) grouped by similar concentration.

## Abbreviations

AA	Antimycin A
ANOVA	Analysis of Variance
ATP	Adenosine Triphosphate
BBB	Blood-Brain Barrier
DMEM	Dulbecco’s Modified Eagle Medium
DMSO	Dimethyl Sulfoxide
ECAR	Extracellular Acidification Rate
FBS	Fetal Bovine Serum
FCCP	Carbonyl cyanide 4-(trifluoromethoxy)phenylhydrazone
FDA	Food and Drug Administration
GSH	Glutathione
HCA	Hydroxycinnamic Acid
HPPA	Hydroxyphenylpropanoic acid
HEPES	2-[4-(2-hydroxyethyl)piperazin-1-yl]ethanesulfonic acid
IQR	Interquartile Range
OCR	Oxygen Consumption Rate
OS	Oxidative Stress
OXPHOS	Oxidative Phosphorylation
PPh_3_	Triphenylphosphine
RA	Retinoic Acid
ROS	Reactive Oxygen Species
ROT	Rotenone
SRB	Sulforhodamine B
TMRM	Tetramethylrhodamine methyl ester perchlorate
TPA	12-O-tetradecanoylphorbol-13-acetate
TPP^+^	Triphenylphosphonium cation
