# Supplementary material for: Cytotoxicity and Mitochondrial Effects of Phenolic and Quinone-Based Mitochondria-Targeted and Untargeted Antioxidants on Human Neuronal and Hepatic Cell Lines: A Comparative Analysis
Source: Biomolecules. 2021 Oct 29;11(11):1605. doi: 10.3390/biom11111605 (PMC8615458; doi:10.3390/biom11111605)
Supplement: Supplementary file 1 [file biomolecules-11-01605-s001.zip › biomolecules-1403652-supplementary.pdf]

# Title

Cytotoxicity and mitochondrial effects of phenolic and quinone based mitochondria-targeted and untargeted antioxidants on human neuronal and hepatic cell lines; a comparative analysis


# Authors list

Carlos Fernandes[1*], Afonso J. C. Videira[1*], Caroline D. Veloso[1*], Sofia Benfeito[2*], Pedro Soares[2], João D. Martins[1], Beatriz Gonçalves[1], José F. S. Duarte[1], António M.S. Santos[1], Paulo J. Oliveira[3], Fernanda Borges[2], José Teixeira[1,3], Filomena SG Silva[1]

# Affiliations

*[1]Mitotag, Biocant Park, Parque Tecnológico de Cantanhede, Núcleo 04, Lote 4, (3060-197) Cantanhede*

*[2]CIQUP/Department of Chemistry and Biochemistry, Faculty of Sciences, University of Porto, (4169-007) Porto, Portugal*

*[3]CNC-Center for Neuroscience and Cell Biology, CIBB - Centre for Innovative Biomedicine and Biotechnology, University of Coimbra, (3004-504) Coimbra, Portugal*

## Corresponding authors:

carlosfernandes@mitotag.com

filomenasilva@mitotag.com

*These authors contributed equally to this work.

# Methods

1. General synthetic procedure for synthesis of alkyl-TPP derivatives

The synthesis of alkyl-TPP derivatives was based on the synthetic strategy already described by Skulachev et al. [44] with few modifications.

Briefly, triphenylphosphine (1.2 eq.) was added to a 2-5 mL glass vial with 1-bromo-hexane, -octane or -decane (0.5 mL; 1 eq.), taking into account the final desired alkyl-TPP derivative. The reaction mixture was stirred and heated to 130 ºC for 24 h under argon atmosphere. After that, the residue was dissolved in dichloromethane and purified by silica gel flash chromatography using dichloromethane:methanol (9:1) as mobile phase. The fractions containing the intended compound were then collected, and the solvent evaporated to dryness.

Hexyltriphenylphosphonium bromide (TPP-C6). Yield = 95 %. $^{1}$H NMR (400 MHz, $CDCl_3$): δ = 0.82 (3H, t, J = 7.1 Hz, H(6)), 1.16 – 1.33 (4H, m, H(4), H(5)), 1.54 – 1.72 (4H, m, H(2), H(3)), 3.69 – 3.84 (2H, m, H(1)), 7.63 – 8.00 (15H, m, H(2′) – H(6′)). $^{13}$C NMR (101 MHz, $CDCl_3$): δ = 14.0 (C(6)), 22.2 (C(5)), 22.6 (d, JCP = 4.6 Hz, C(3)), 22.8 (d, JCP = 49.7 Hz, C(1)), 30.1 (d, JCP = 15.5 Hz, C(2)), 31.3 (d, JCP = 1.0 Hz, C(4)), 118.4 (d, JCP = 85.8 Hz, 3 × C(1′)), 130.5 (d, JCP = 12.5 Hz, 3 × C(3′) and 3 × C(5′)), 133.7 (d, JCP = 10.0 Hz, 3 × C(2′) and 3 × C(6′)), 135.0 (d, JCP = 3.0 Hz, 3 × C(4′)).

Octyltriphenylphosphonium bromide (TPP-C8). Yield = 68 %. $^{1}$H NMR (400 MHz, $CDCl_3$): $\delta$ = 0.83 (3H, t, J = 6.9 Hz, H(8)), 1.13 – 1.32 (8H, m, H(4) – H(7)), 1.56 – 1.70 (4H, m, H(2), H(3)), 3.45 – 3.97 (2H, m, H(1)), 7.62 – 7.94 (15H, m, H(2′) – H(6′)). $^{13}$C NMR (101 MHz, $CDCl_3$): $\delta$ = 14.1 (C(8)), 22.5 (C(7)), 22.7 (d, JCP = 4.6 Hz, C(3)), 22.8 (d, JCP = 49.5 Hz, C(1)), 28.8 (C(6)), 29.2 (d, JCP = 1.0 Hz, C(4)), 30.4 (d, JCP = 15.5 Hz, C(2)), 31.7 (C(5)), 118.5 (d, JCP = 85.8 Hz, 3 × C(1′)), 130.5 (d, JCP = 12.5 Hz, 3 × C(3′) and 3 × C(5′)), 133.7 (d, JCP = 10.0 Hz, 3 × C(2′) and 3 × C(6′)), 135.0 (d, JCP = 3.0 Hz, 3 × C(4′)).

Decyltriphenylphosphonium bromide (TPP-C10). Yield = 92 %. $^{1}$H NMR (400 MHz, $CDCl_3$): $\delta$ = 0.85 (3H, t, J = 7.0 Hz, H(10)), 1.13 – 1.32 (12H, m, H(4) – H(9)), 1.55 – 1.69 (4H, m, H(2), H(3)), 3.68 – 3.91 (2H, m, H(1)), 7.63 – 7.96 (15H, m, H(2′) – H(6′)). $^{13}$C NMR (101 MHz, $CDCl_3$): $\delta$ =

14.1 (C(10)), 22.5 (C(9)), 22.7 (d, JCP = 4.7 Hz, C(3)), 22.8 (d, JCP = 49.5 Hz, C(1)), 29.2 (C(7)), 29.2 (C(5), C(6)), 29.5 (C(4)), 30.4 (d, JCP = 15.5 Hz, C(2)), 31.8 (C(8)), 118.5 (d, JCP = 85.8 Hz, 3 × C(1′)), 130.5 (d, JCP = 12.5 Hz, 3 × C(3′) and 3 × C(5′)), 133.7 (d, JCP = 9.9 Hz, 3 × C(2′) and 3 × C(6′)), 135.0 (d, JCP = 3.0 Hz, 3 × C(4′)).

# Supplementary Figures and Legends

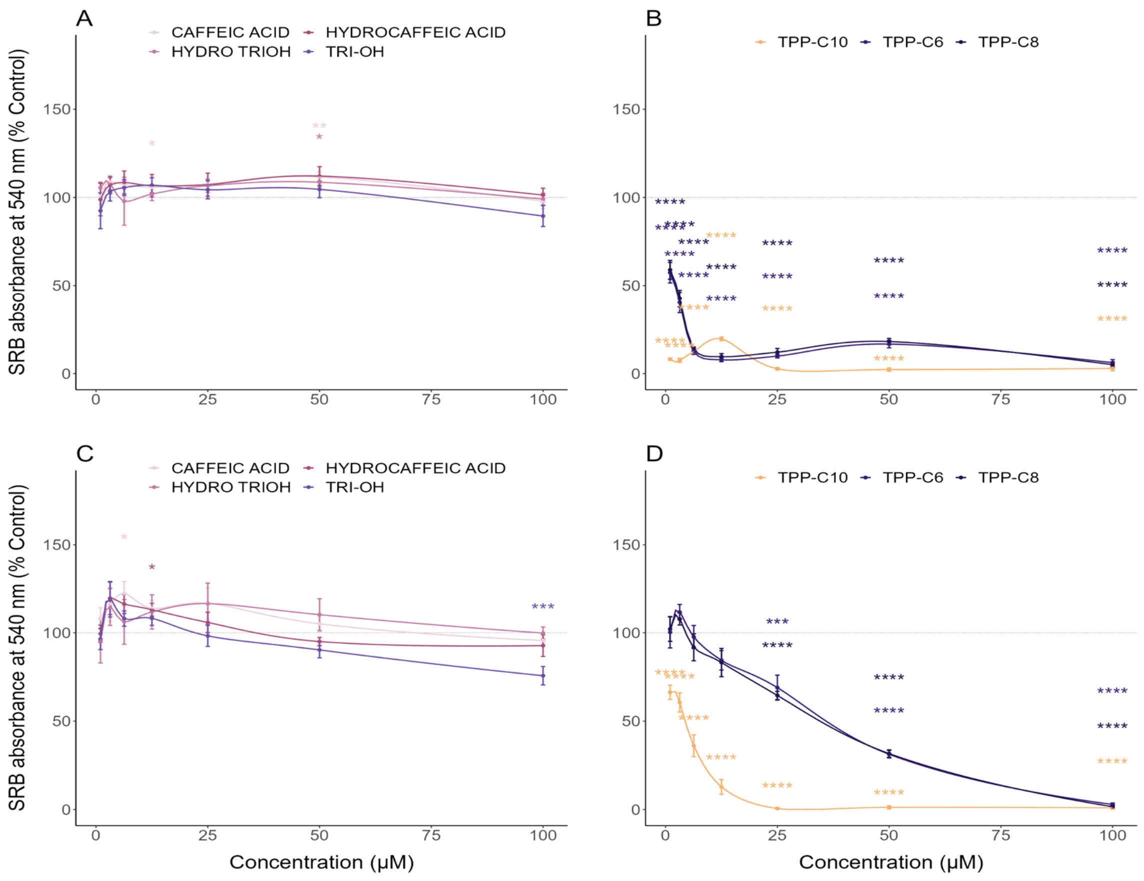


**Figure S1** Effect of parental antioxidants and alkylTPP compounds on cell mass. Human caucasian hepatocyte carcinoma (HepG2, A and B) and differentiated human neuroblastoma (SH-SY5Y, C and D) were treated with increasing concentrations of the different molecules for a period of 48 h and cellular mass was evaluated using Sulforhodamine B (SRB) assay. Data are the mean ± SE of four independent experiments and the results are expressed as percentage of the control. Statistically significant differences between control (CTL) and treated groups were evaluated as described in the Materials and Methods. ****p<0.0001, ***p<0.001, **p<0.01 and *p<0.05 compared to the respective control (CTL).

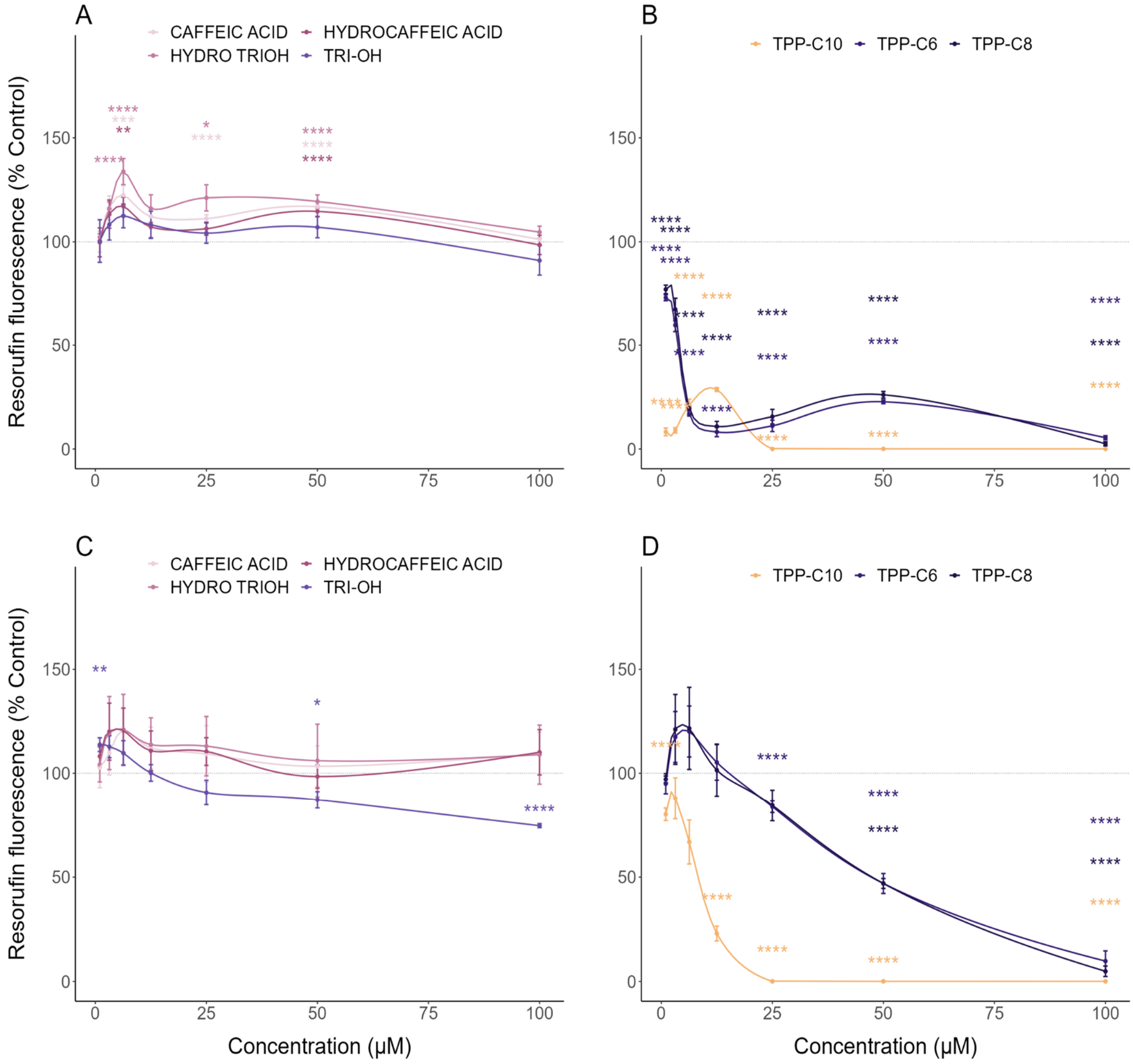


**Figure S2** Effect of parental antioxidants and alkylTPP compounds on metabolic activity. Human caucasian hepatocyte carcinoma (HepG2, A and-B) and differentiated human neuroblastoma (SH-SY5Y, C and D) were treated with increasing concentrations of the different molecules for a period of 48 h and metabolic activity was evaluated using resazurin reduction assay. Data are the mean ± SE of four independent experiments and the results are expressed as percentage of the control (control=100%). Statistically significant differences between control (CTL) and treated groups were evaluated using a t-test. ****$p<0.0001$, ***$p<0.001$, **$p<0.01$ and *$p<0.05$ compared to the respective control (CTL).

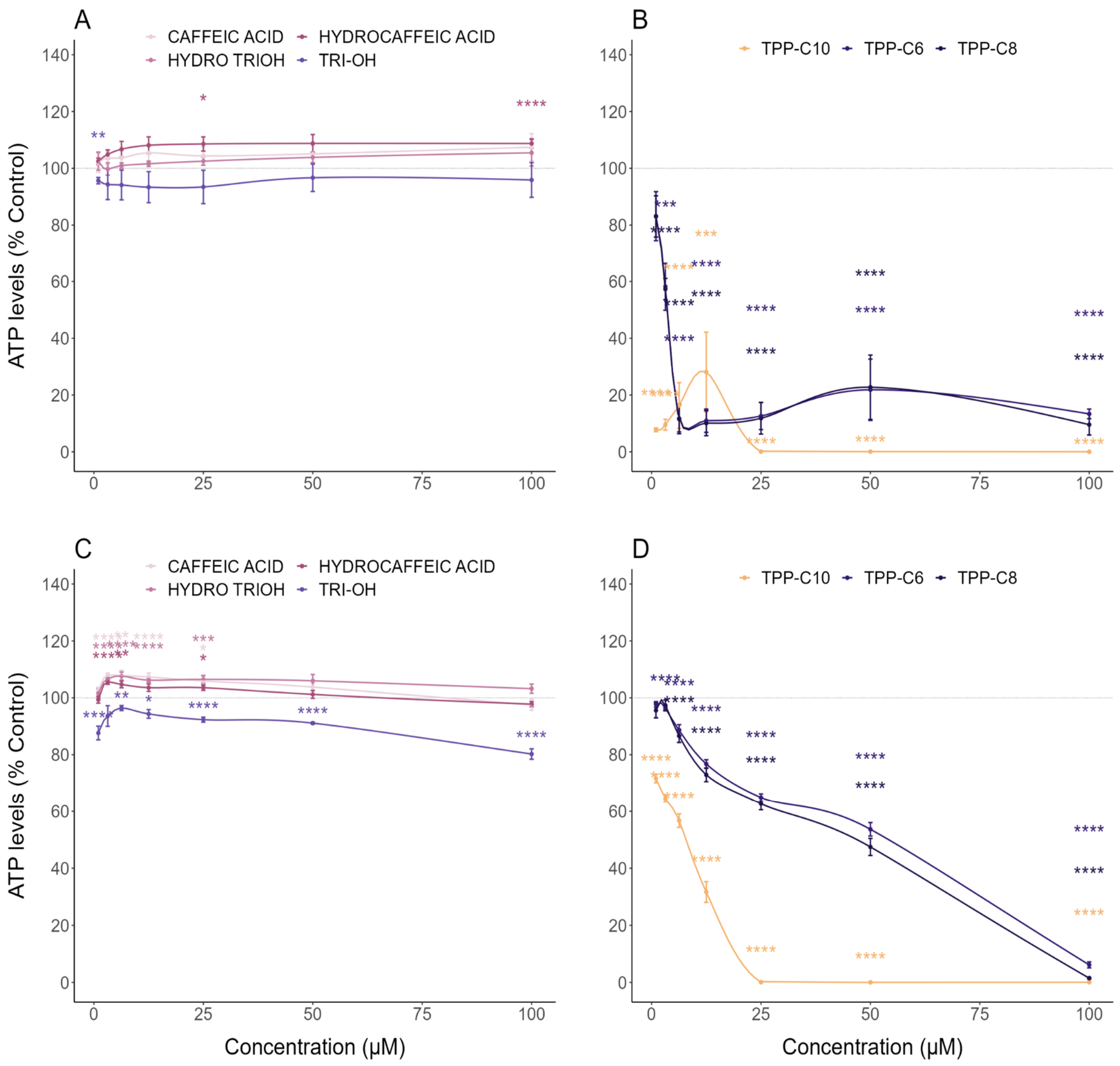


**Figure S3** Effect of parental antioxidants and alkylTPP compounds on ATP intracellular concentrations. Human caucasian hepatocyte carcinoma (HepG2, A and B) and differentiated human neuroblastoma (SH-SY5Y C and D) were treated with increasing concentrations of the different molecules for a period of 48 h, and intracellular ATP levels were evaluated using CellTiter-Glo® Luminescent Cell Viability Assay. Data are the mean ± SE of three independent experiments and the results are expressed as a percentage of the control (control=100%). Statistically significant differences between control (CTL) and treated groups were evaluated using a t-test. ****$p<0.0001$, ***$p<0.001$, **$p<0.01$ and *$p<0.05$ compared to the respective control (CTL).

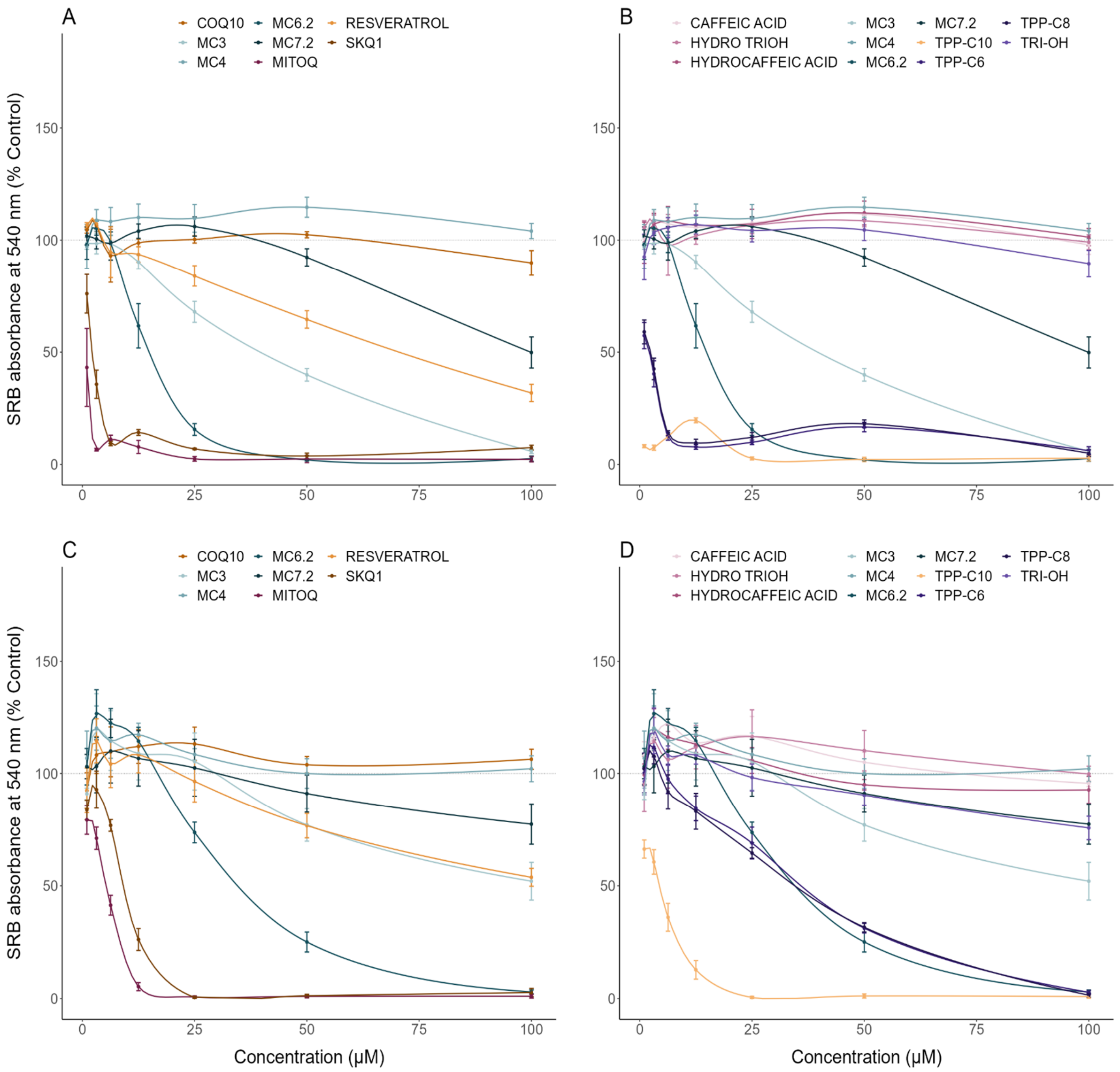


**Figure S4** Comparison between the effects of MitoCINs, quinone-based mitochondria-targeted, non-targeted antioxidants, parental antioxidants and alkylTPP compounds on cell mass. Human caucasian hepatocyte carcinoma (HepG2, A and B) and differentiated human neuroblastoma (SH-SY5Y, C and D) were treated with increasing concentrations of the different compounds for 48 h and cellular mass was evaluated using the Sulforhodamine B (SRB) assay. Data are the mean ± SE of four independent experiments and the results are expressed as a percentage of the control (considered as 100%). Statistically significant differences between treated groups were evaluated as described in the Materials and Methods. For the sake of simplifying the figure, the different statistical comparisons in this figure are present in Tables 1-8 in the external document file (link: https://bit.ly/3mNTtsz).

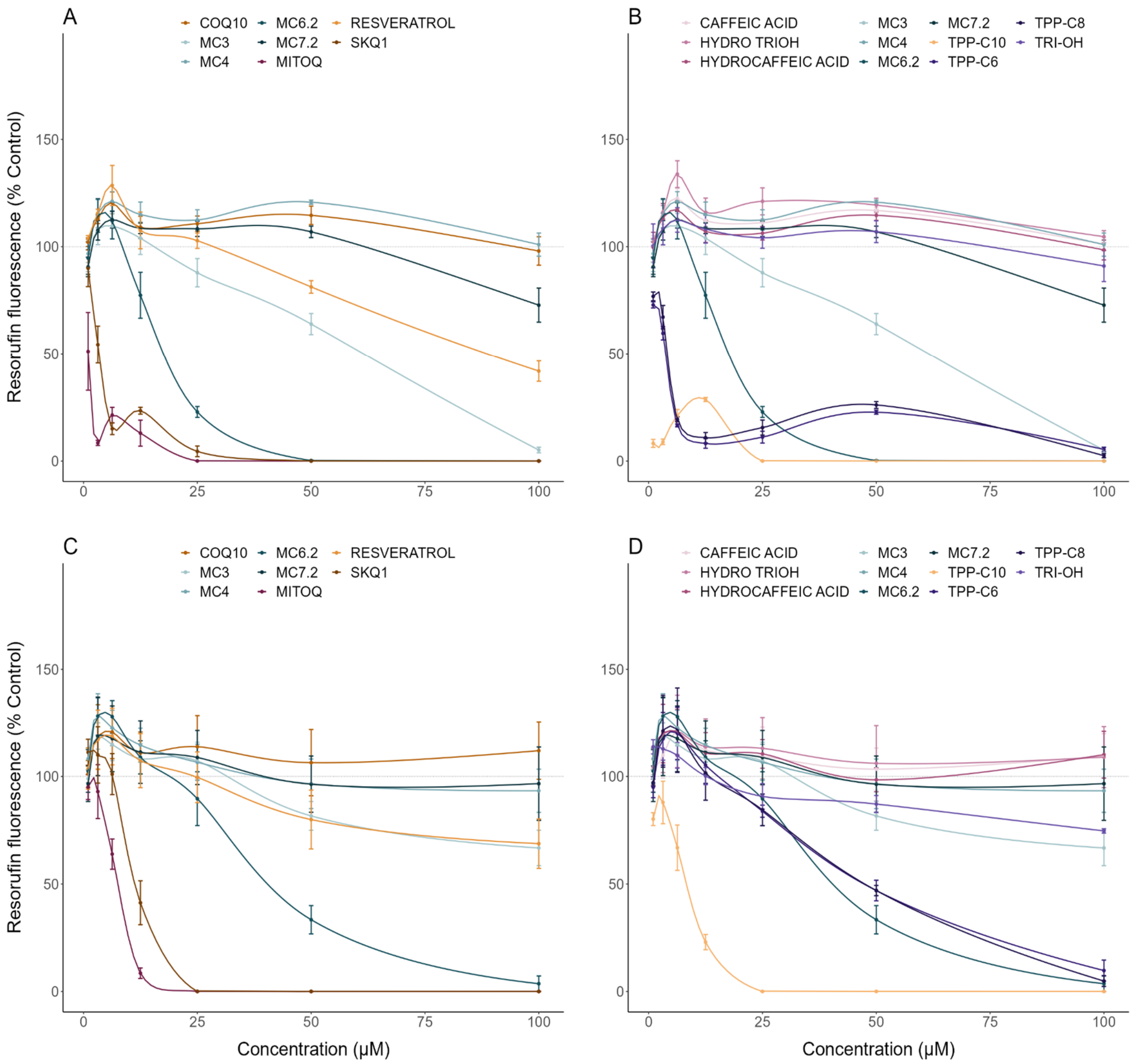


**Figure S5** Comparison between the effects of MitoCINs, quinone-based mitochondria-targeted, non-targeted antioxidants, parental antioxidants and alkylTPP compounds on metabolic activity. Human caucasian hepatocyte carcinoma (HepG2, A and B) and differentiated human neuroblastoma (SH-SY5Y, C and D) were treated with increasing concentrations of the different molecules for 48 h and metabolic activity was evaluated using resazurin reduction assay. Data are the mean ± SE of four independent experiments and the results are expressed as percentage of the control (considered as 100%). Statistically significant differences between treated groups were evaluated as described in the Materials and Methods. For the sake of simplifying the figure, the different statistical comparisons in this figure are present in Tables 9-16 in the external document file (linK: https://bit.ly/3mNTtsz).

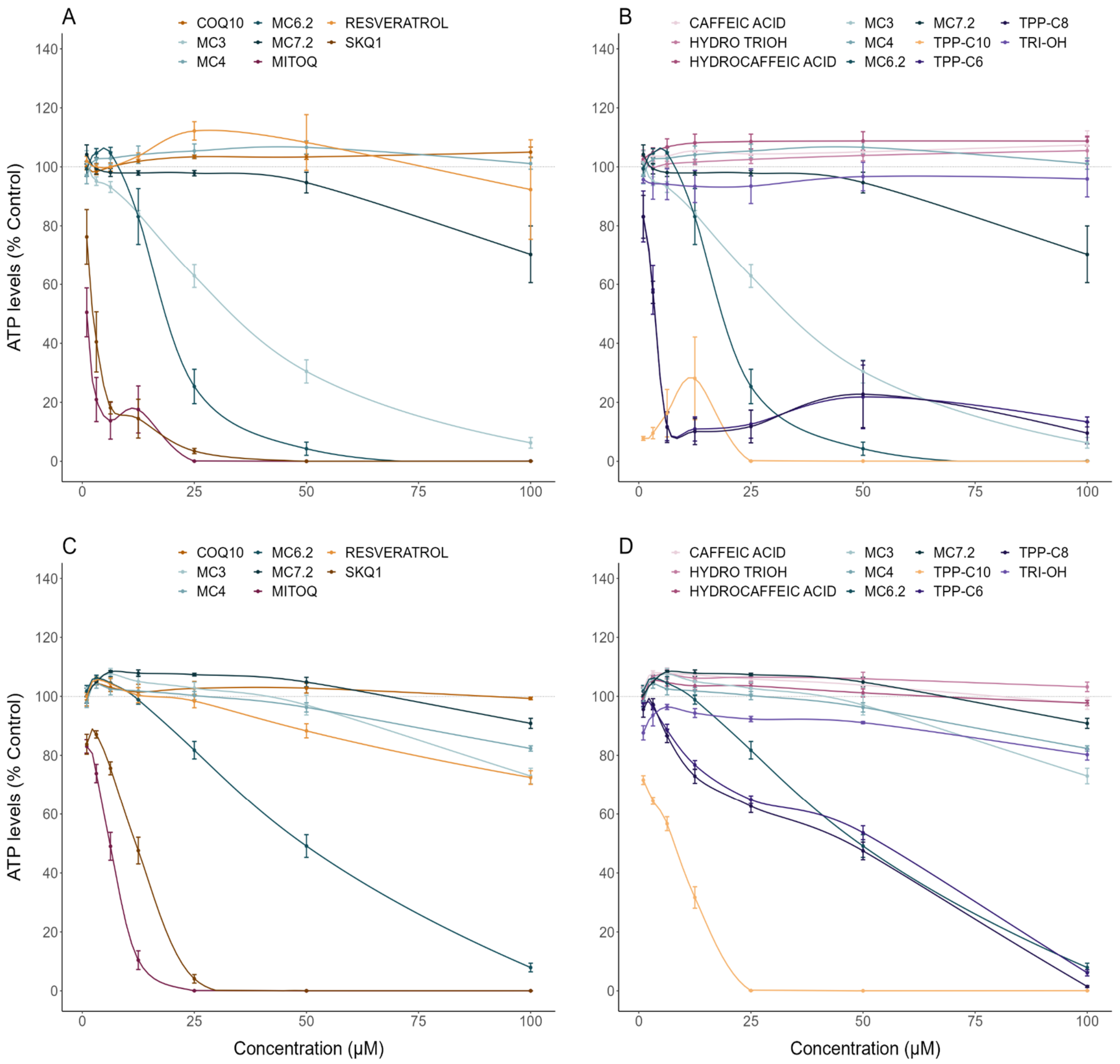


**Figure S6** Comparison between the effects of MitoCINs, quinone-based mitochondria-targeted, non-targeted antioxidants, parental antioxidants and alkylTPP compounds on ATP intracellular concentrations. Human caucasian hepatocyte carcinoma (HepG2, A and B) and differentiated human neuroblastoma (SH-SY5Y, C and D) were treated with increasing concentrations of the different molecules for 48 h, and intracellular ATP levels were evaluated using the CellTiter-Glo® Luminescent Cell Viability Assay. Data are the mean ± SE of three independent experiments and the results are expressed as a percentage of the control (control=100%). Statistically significant differences between treated groups were evaluated as described in the Materials and Methods. For the sake of simplifying the figure, the different statistical comparisons in this figure are present in Tables 17-24 in the external document file (linK: https://bit.ly/3mNTtsz).

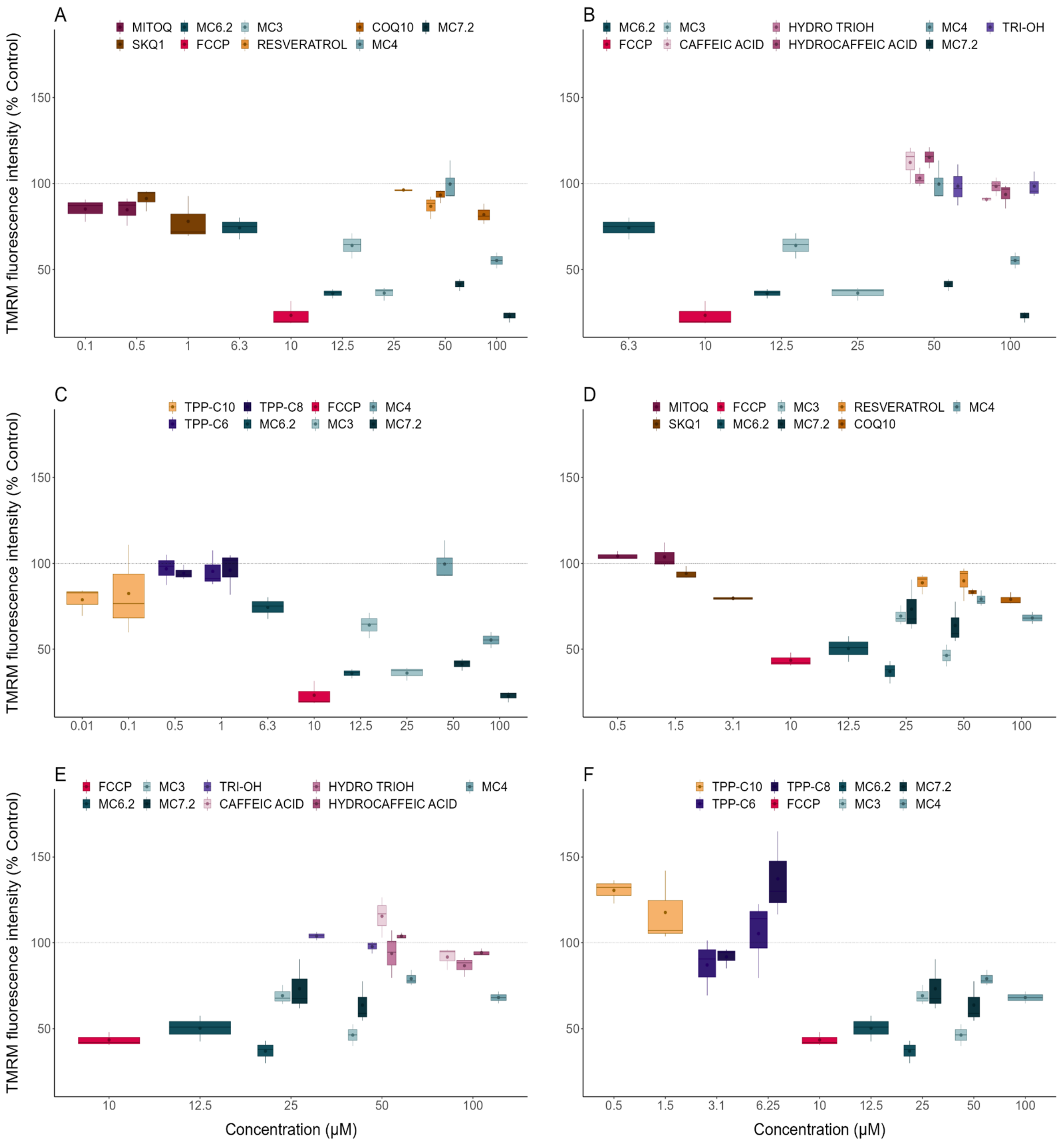


**Figure S7** Comparison between the effects of MitoCINs, quinone-based mitochondria-targeted, non-targeted antioxidants, parental antioxidants and alkylTPP compounds on TMRM intensity fluorescence. Human caucasian hepatocyte carcinoma (HepG2, A, B and C) and differentiated human neuroblastoma (SH-SY5Y, D, E and F) were treated with increasing concentrations of the different molecules for 48 h and mitochondrial membrane potential was indirectly evaluated using TMRM fluorescence intensity. Data are the mean ± SE of three independent experiments and the results are expressed as percentage of the control (control=100%). Statistically significant differences between treated groups were evaluated as described in the Materials and Methods. For the sake of simplifying the figure, the different statistical comparisons in this figure are present in Tables 25-32 in the external document file (linK: https://bit.ly/3mNTtsz).

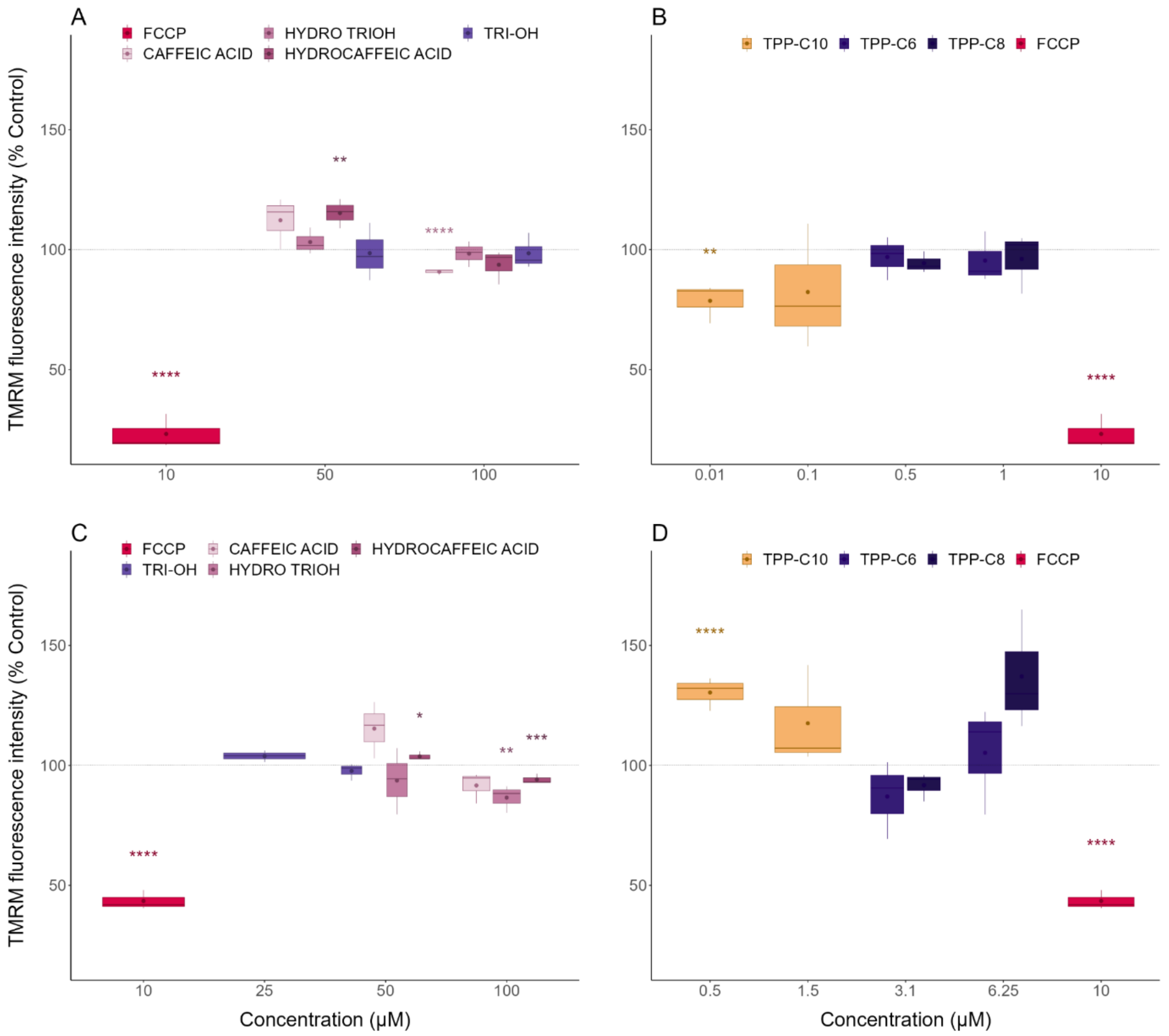


**Figure S8** Effect of parental antioxidants and alkylTPP compounds on TMRM intensity fluorescence. Human caucasian hepatocyte carcinoma (HepG2, A and B) and differentiated human neuroblastoma (SH-SY5Y, C and D) were treated with increasing concentrations of the different molecules for a period of 48 h and mitochondrial membrane potential was indirectly evaluated using TMRM fluorescence intensity. Data are the mean± SE of three independent experiments and the results are expressed as percentage of the control (control=100%). Statistically significant differences between control (CTL) and treated groups were evaluated using a t-test. ****$p<0.0001$, ***$p<0.001$, **$p<0.01$ and *$p<0.05$ compared to the respective control (CTL).

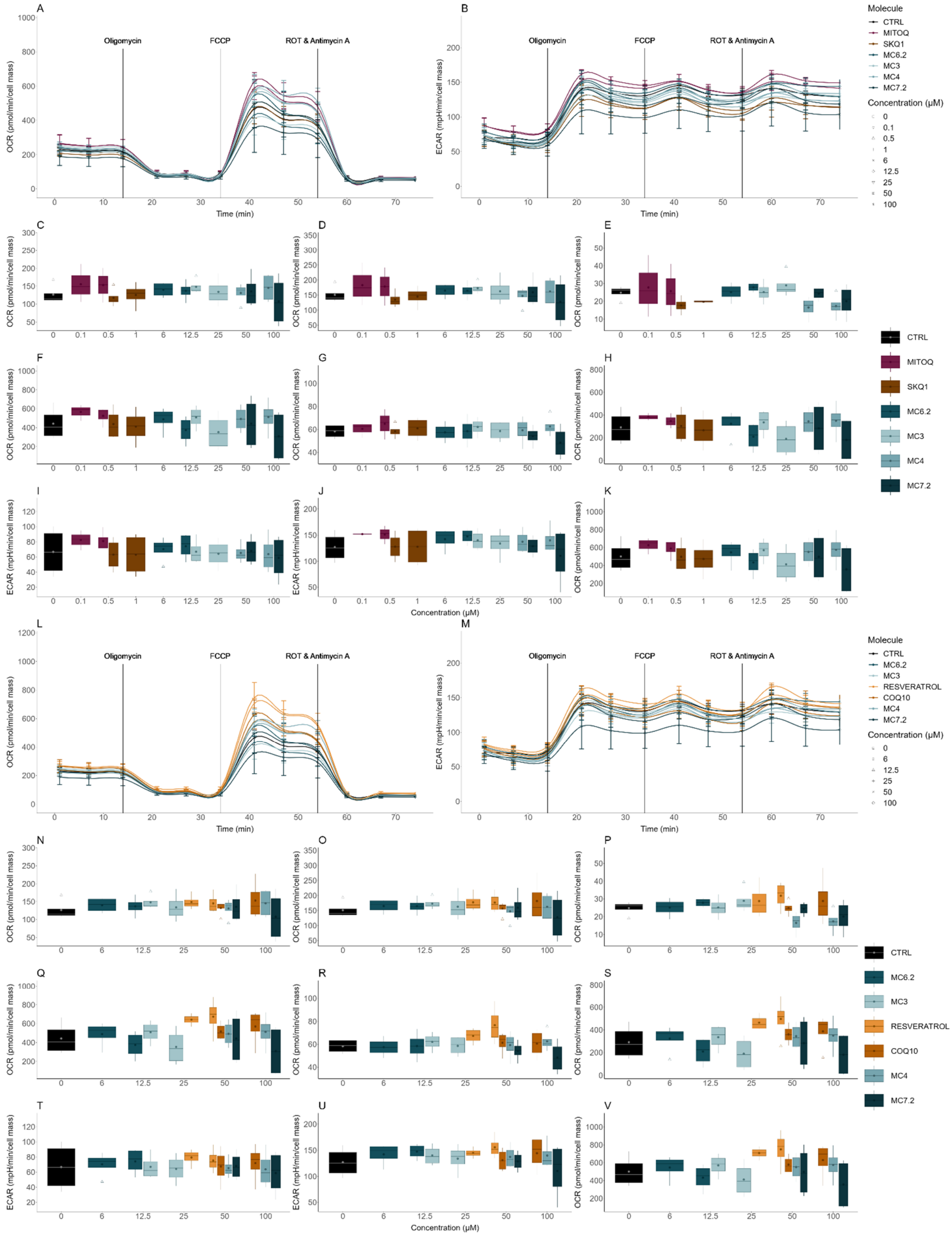


**Figure S9** Comparison between the effects of MitoCINs, quinone-based mitochondria-targeted and non-targeted antioxidants on oxygen consumption rate (OCR) and extracellular acidification rate (ECAR) in human caucasian hepatocyte carcinoma (HepG2) cells. OCR- and ECAR-associated parameters were assessed with the Seahorse XFe96 Extracellular Flux Analyzer. OCR (A, L) and ECAR (B, M) were assessed in HepG2 cells treated with increasing concentrations of the different molecules for 48 h. Several OCR parameters were evaluated: ATP production-linked OCR (C, N), basal respiration (D, O), proton leak-based OCR (E, P), maximal respiration (F, Q),

non-mitochondrial respiration (G, R), spare respiratory capacity (H, S) and ECAR parameters were also evaluated, including basal ECAR (I, T), stressed ECAR (J, U) and stressed OCR (K, V). Data are the mean± SE of four independent experiments and the results are expressed in interquartile range (Q1-Q3) together with the (–) median. Statistically significant differences between treated groups were evaluated as described in the Materials and Methods. For the sake of simplifying the figure, the different statistical comparisons in this figure are present in Tables 33-50 in the external document file (linK: https://bit.ly/3mNTtsz).

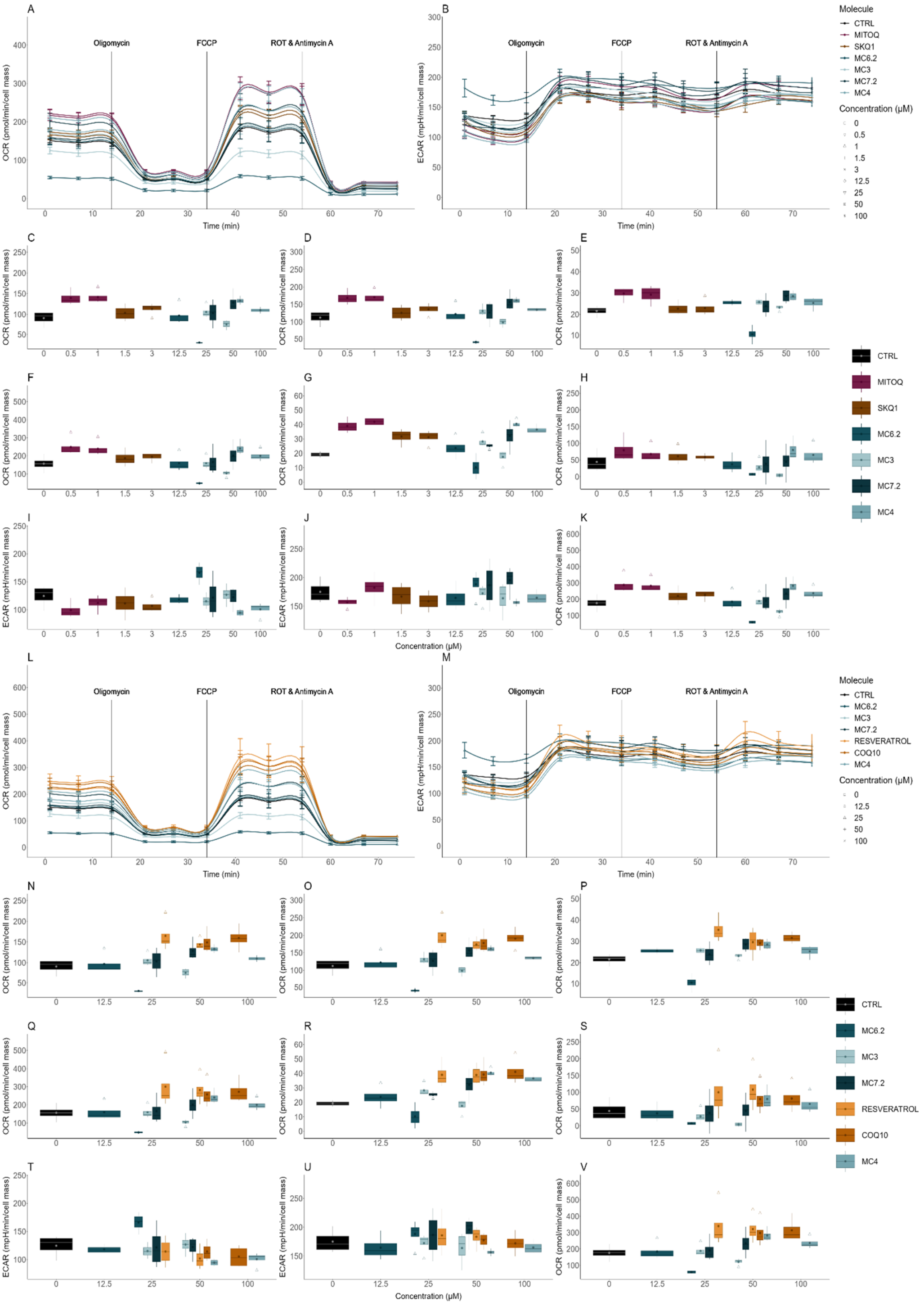

**Figure S10** Comparison between the effects of MitoCINs, quinone-based mitochondria-targeted and non-targeted antioxidants on oxygen consumption rate (OCR) and extracellular acidification rate (ECAR) in differentiated human neuroblastoma (SH-SY5Y) cells. OCR- and ECAR-associated parameters were assessed with the Seahorse XFe96 Extracellular Flux Analyzer. OCR (A, L) and ECAR (B, M) were assessed in SH-SY5Y cells treated with increasing concentrations of the different molecules for 48 h. Several OCR parameters were evaluated: ATP production-linked OCR (C, N), basal respiration (D, O), proton leak-based OCR (E, P), maximal respiration (F, Q), non-mitochondrial respiration (G, R), spare respiratory capacity (H, S) and ECAR parameters were also evaluated, including basal ECAR (I, T), stressed ECAR (J, U) and stressed OCR (K, V). Data are the mean± SE of four independent experiments, and the results are expressed in interquartile range (Q1-Q3) together with the (−) median. Statistically significant differences between treated groups were evaluated as described in the Materials and Methods. For the sake of simplifying the figure, the different statistical comparisons in this figure are present in Tables 51-68 in the external document file (linK: https://bit.ly/3mNTtsz).

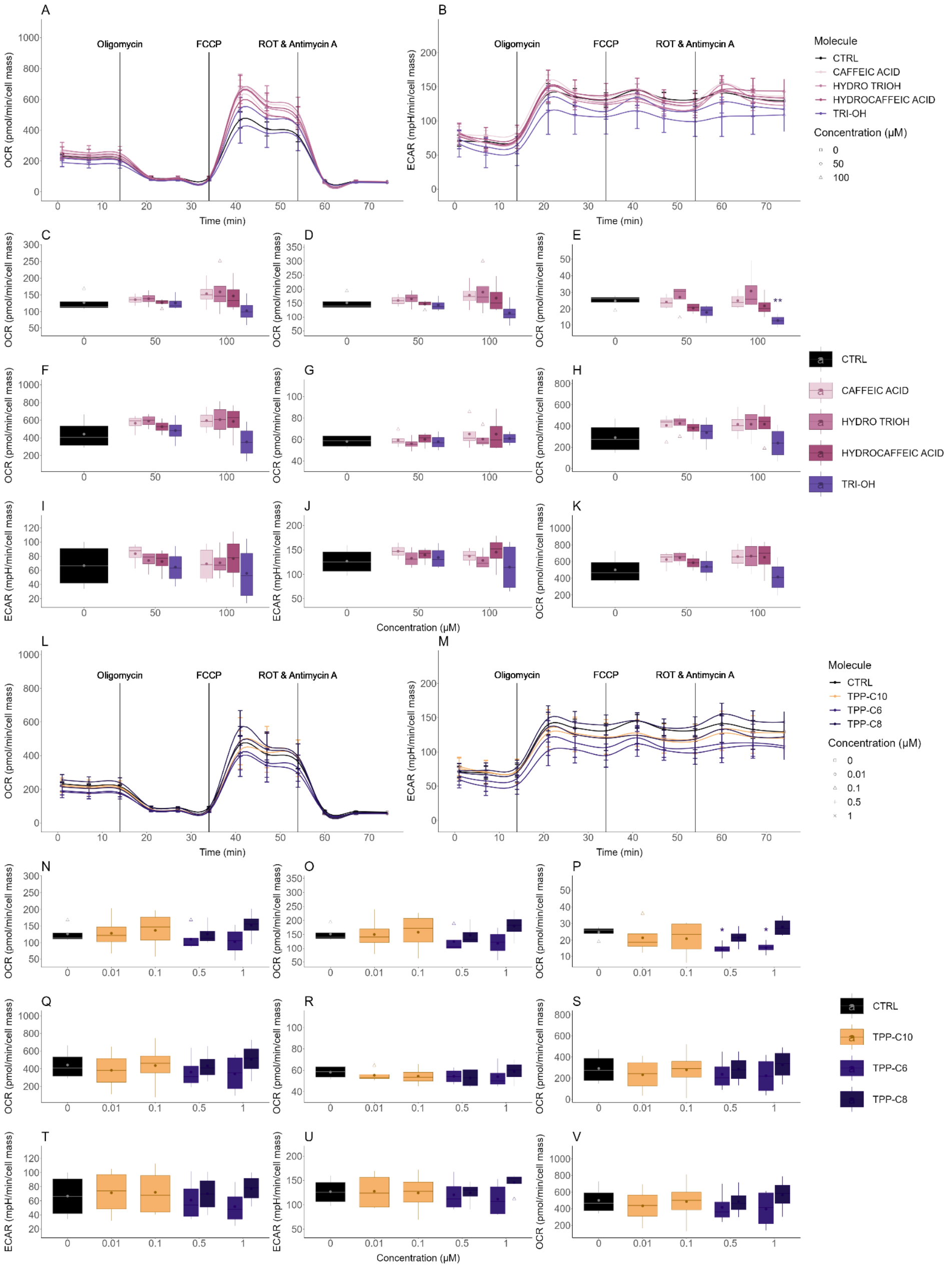


**Figure S11** Effect of parental antioxidants and alkylTPP compounds on oxygen consumption rate (OCR) and extracellular acidification rate (ECAR). OCR- and ECAR-associated parameters were assessed with the Seahorse XFe96 Extracellular Flux Analyzer. OCR (A, L) and ECAR (B, M) were assessed in human caucasian hepatocyte carcinoma (HepG2) cells treated with increasing concentrations of the different molecules for a period of 48 h. Several OCR parameters were evaluated: ATP production-linked OCR (C, N), basal respiration (D, O), proton leak-based OCR (E, P), maximal respiration (F, Q), non-mitochondrial respiration (G, R), spare respiratory capacity (H, S) and ECAR parameters were also evaluated, including basal ECAR (I, T), stressed ECAR (J, U) and stressed OCR (K, V). Data are the mean± SE of four independent experiments and the results are expressed in interquartile range (Q1-Q3) together with the (–) median. Statistically significant differences between control (CTL) and treated groups were evaluated using a t-test.**p<0.01 and *p<0.05 compared to the respective control (CTL).

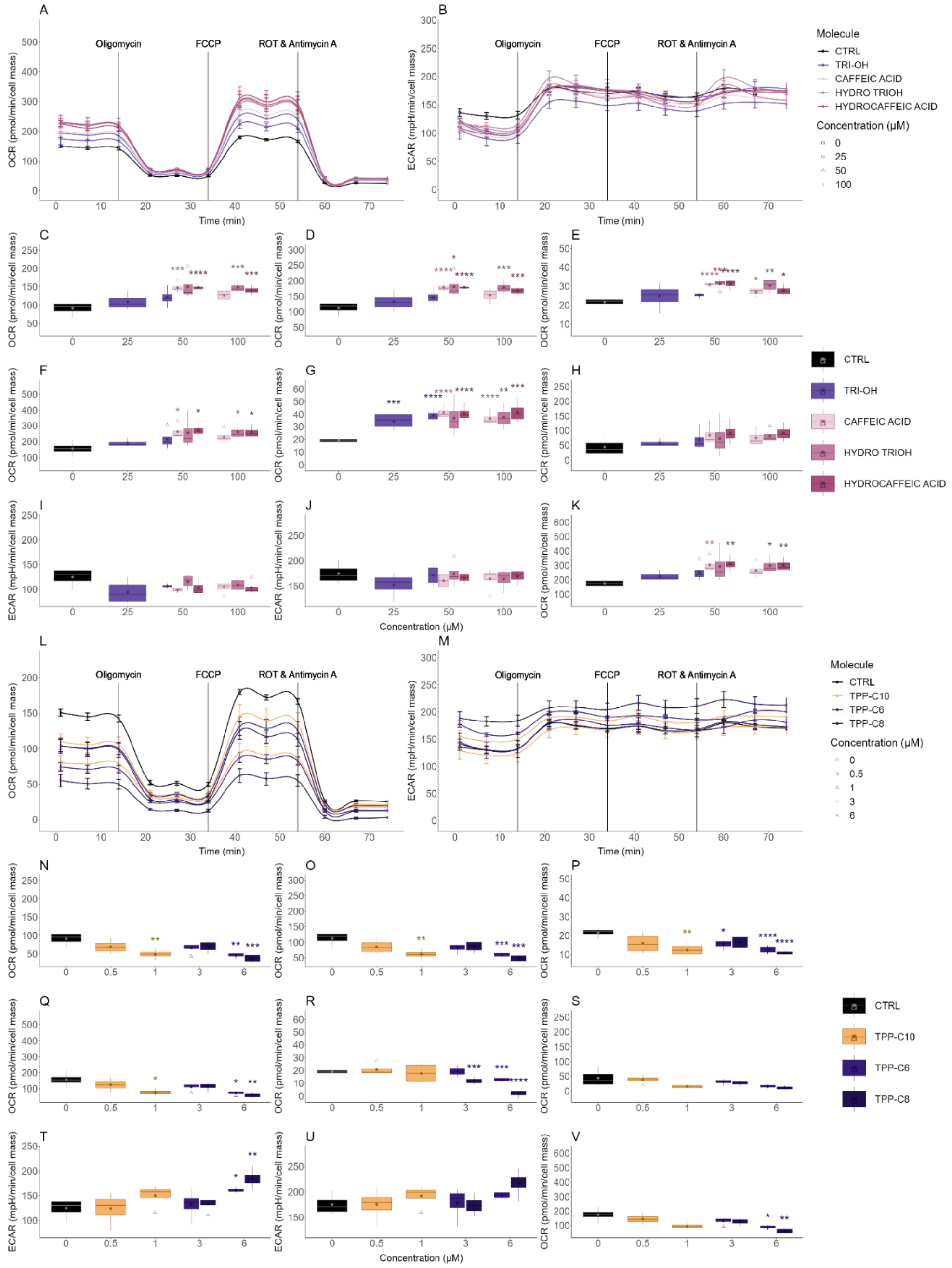


**Figure S12** Effect of parental antioxidants and alkylTPP compounds on oxygen consumption rate (OCR) and extracellular acidification rate (ECAR). OCR- and ECAR-associated parameters were assessed with the Seahorse XFe96 Extracellular Flux Analyzer. OCR (A, L) and ECAR (B, M) were assessed in differentiated human neuroblastoma (SH-SY5Y) cells treated with increasing concentrations of the different molecules for a period of 48 h. Several OCR parameters were evaluated: ATP production-linked OCR (C, N), basal respiration (D, O), proton leak-based OCR (E, P), maximal respiration (F, Q), non-mitochondrial respiration (G, R), spare respiratory capacity (H, S) and ECAR parameters were also evaluated, including basal ECAR (I, T), stressed ECAR (J, U) and stressed OCR (K, V). Data are the mean± SE of four independent experiments and the results are expressed in interquartile range (Q1-Q3) together with the (–) median. . Statistically significant differences between control (CTL) and treated groups were evaluated using a t-test. ****p<0.0001, ***p<0.001, **p<0.01 and *p<0.05 compared to the respective control (CTL).

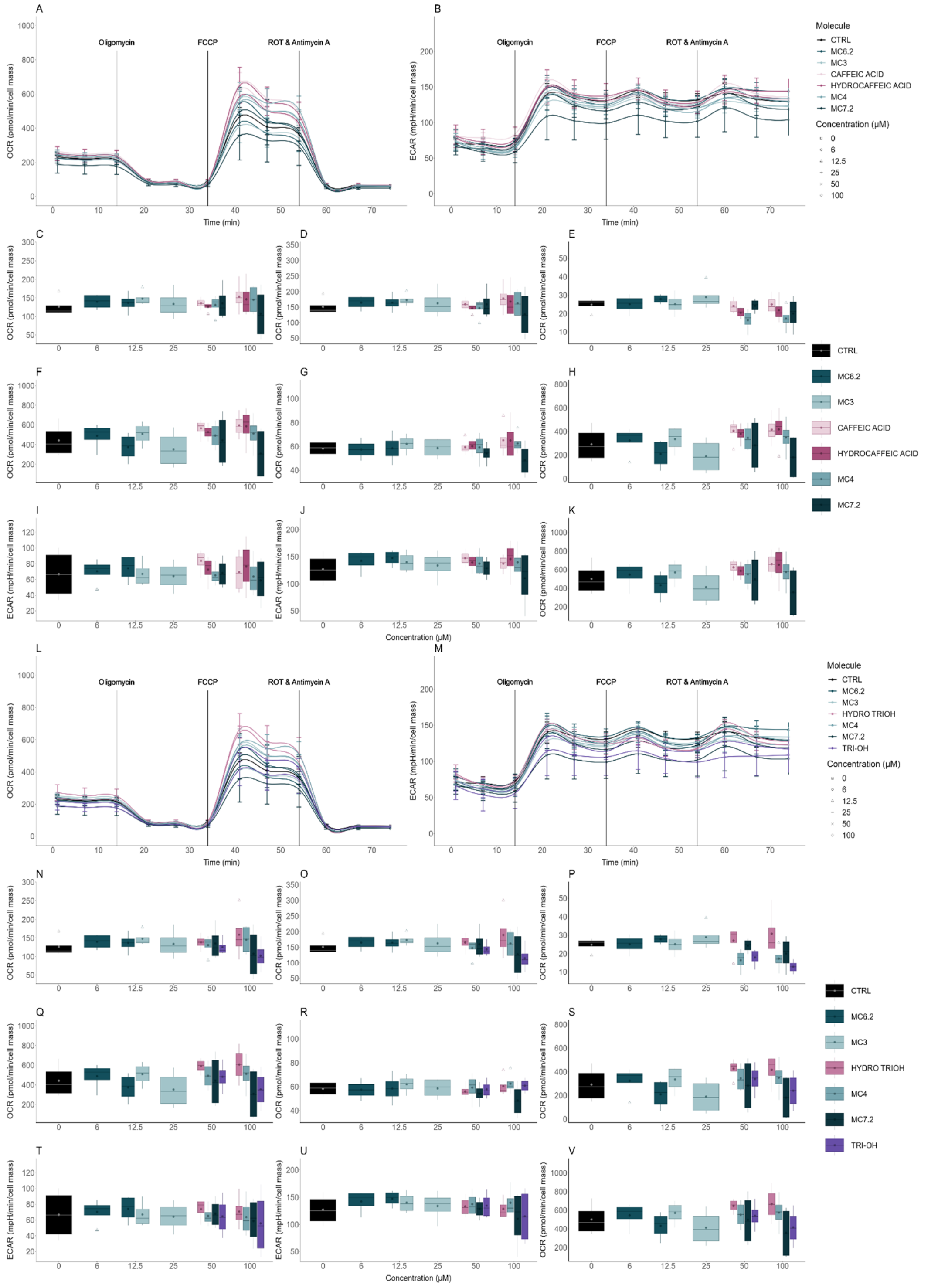

**Figure S13** Comparison between the effects of MitoCINs and parental antioxidants on oxygen consumption rate (OCR) and extracellular acidification rate (ECAR) in human caucasian hepatocyte carcinoma (HepG2) cells. OCR- and ECAR-associated parameters were assessed with the Seahorse XFe96 Extracellular Flux Analyzer. OCR (A, L) and ECAR (B, M) were assessed in HepG2 cells treated with increasing concentrations of the different molecules for 48 h. Several OCR parameters were evaluated: ATP production-linked OCR (C, N), basal respiration (D, O), proton leak-based OCR (E, P), maximal respiration (F, Q), non-mitochondrial respiration (G, R), spare respiratory capacity (H, S) and ECAR parameters were also evaluated, including basal ECAR (I, T), stressed ECAR (J, U) and

stressed OCR (K, V). Data are the mean± SE of four independent experiments and the results are expressed in interquartile range (Q1-Q3) together with the (–) median. Statistically significant differences between treated groups were evaluated as described in the Materials and Methods. For the sake of simplifying the figure, the different statistical comparisons in this figure are present in Tables 69-86 in the external document file (linK: https://bit.ly/3mNTtsz).

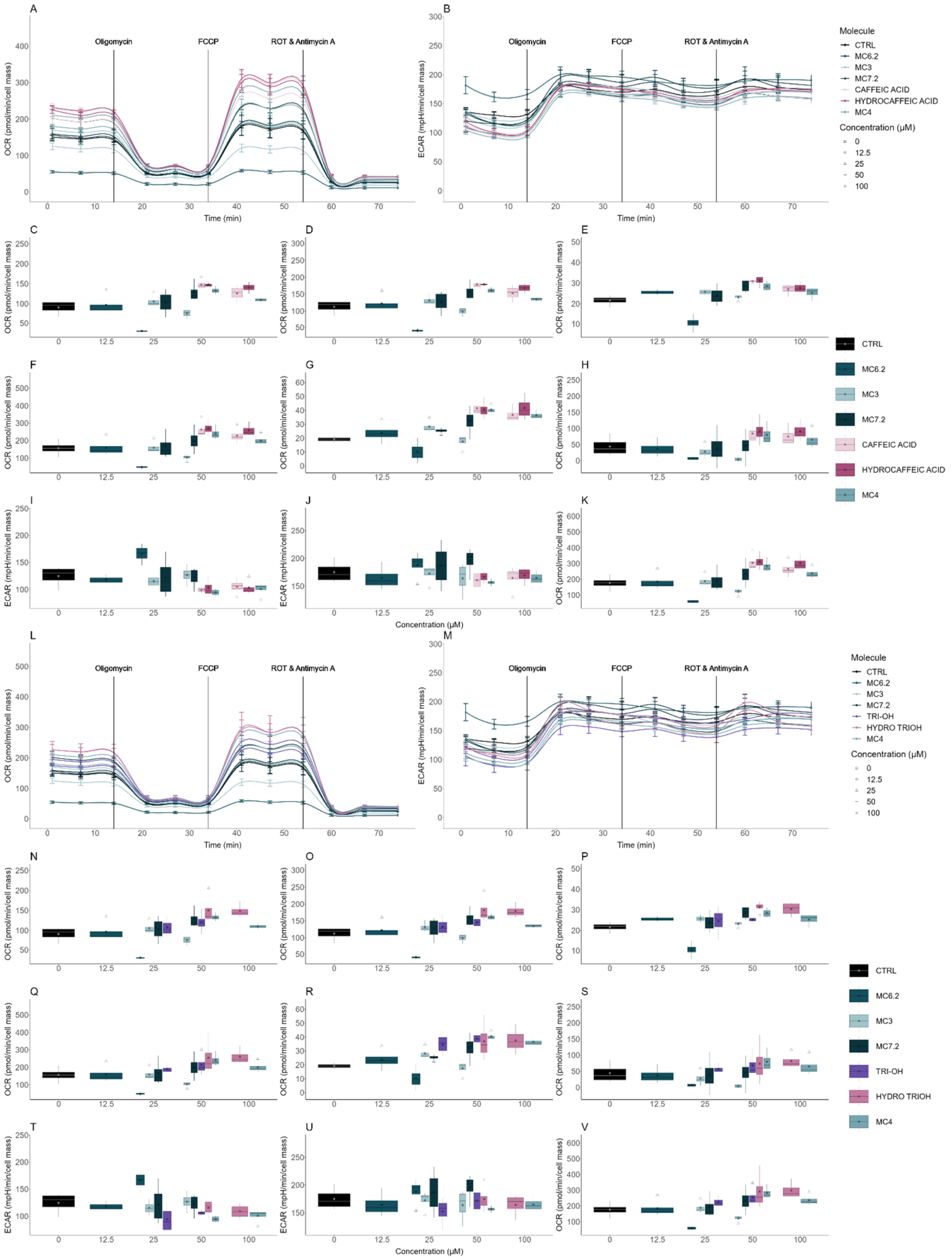


**Figure S14** Comparison between the effects of MitoCINs and parental antioxidants on oxygen consumption rate (OCR) and extracellular acidification rate (ECAR) in differentiated human neuroblastoma (SH-SY5Y) cells. OCR-

and ECAR-associated parameters were assessed with the Seahorse XFe96 Extracellular Flux Analyzer. OCR (A, L) and ECAR (B, M) were assessed in SH-SY5Y cells treated with increasing concentrations of the different molecules for 48 h. Several OCR parameters were evaluated: ATP production-linked OCR (C, N), basal respiration (D, O), proton leak-based OCR (E, P), maximal respiration (F, Q), non-mitochondrial respiration (G, R), spare respiratory capacity (H, S) and ECAR parameters were also evaluated, including basal ECAR (I, T), stressed ECAR (J, U) and stressed OCR (K, V). Data are the mean± SE of four independent experiments and the results are expressed in interquartile range (Q1-Q3) together with the (–) median. Statistically significant differences between treated groups were evaluated as described in the Materials and Methods. For the sake of simplifying the figure, the different statistical comparisons in this figure are present in Tables 87-104 in the external document (linK: https://bit.ly/3mNTtsz).

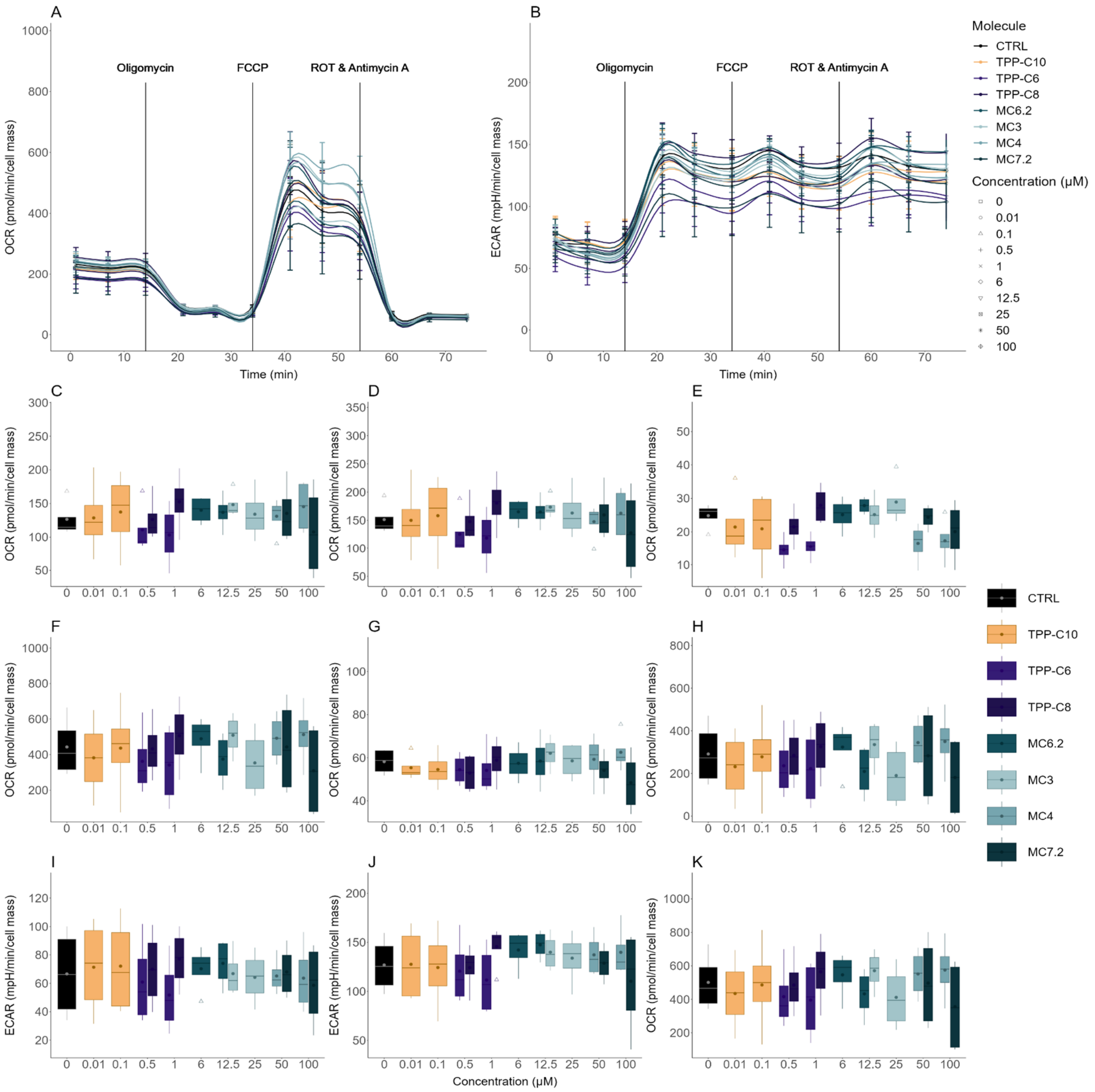


**Figure S15** Comparison between the effects of MitoCINs and alkylTPP compounds on oxygen consumption rate (OCR) and extracellular acidification rate (ECAR) in human caucasian hepatocyte carcinoma (HepG2) cells. OCR-

and ECAR-associated parameters were assessed with the Seahorse XFe96 Extracellular Flux Analyzer. OCR (A) and ECAR (B) were assessed in HepG2 cells treated with increasing concentrations of the different molecules for 48 h. Several OCR parameters were evaluated: ATP production-linked OCR (C), basal respiration (D), proton leak-based OCR (E), maximal respiration (F), non-mitochondrial respiration (G), spare respiratory capacity (H) and ECAR parameters were also evaluated, including basal ECAR (I), stressed ECAR (J) and stressed OCR (K). Data are the mean± SE of four independent experiments and the results are expressed in interquartile range (Q1-Q3) together with the (–) median. Statistically significant differences between treated groups were evaluated as described in the Materials and Methods. For the sake of simplifying the figure, the different statistical comparisons in this figure are present in Tables 69-86 in the external document file (linK: https://bit.ly/3mNTtsz).

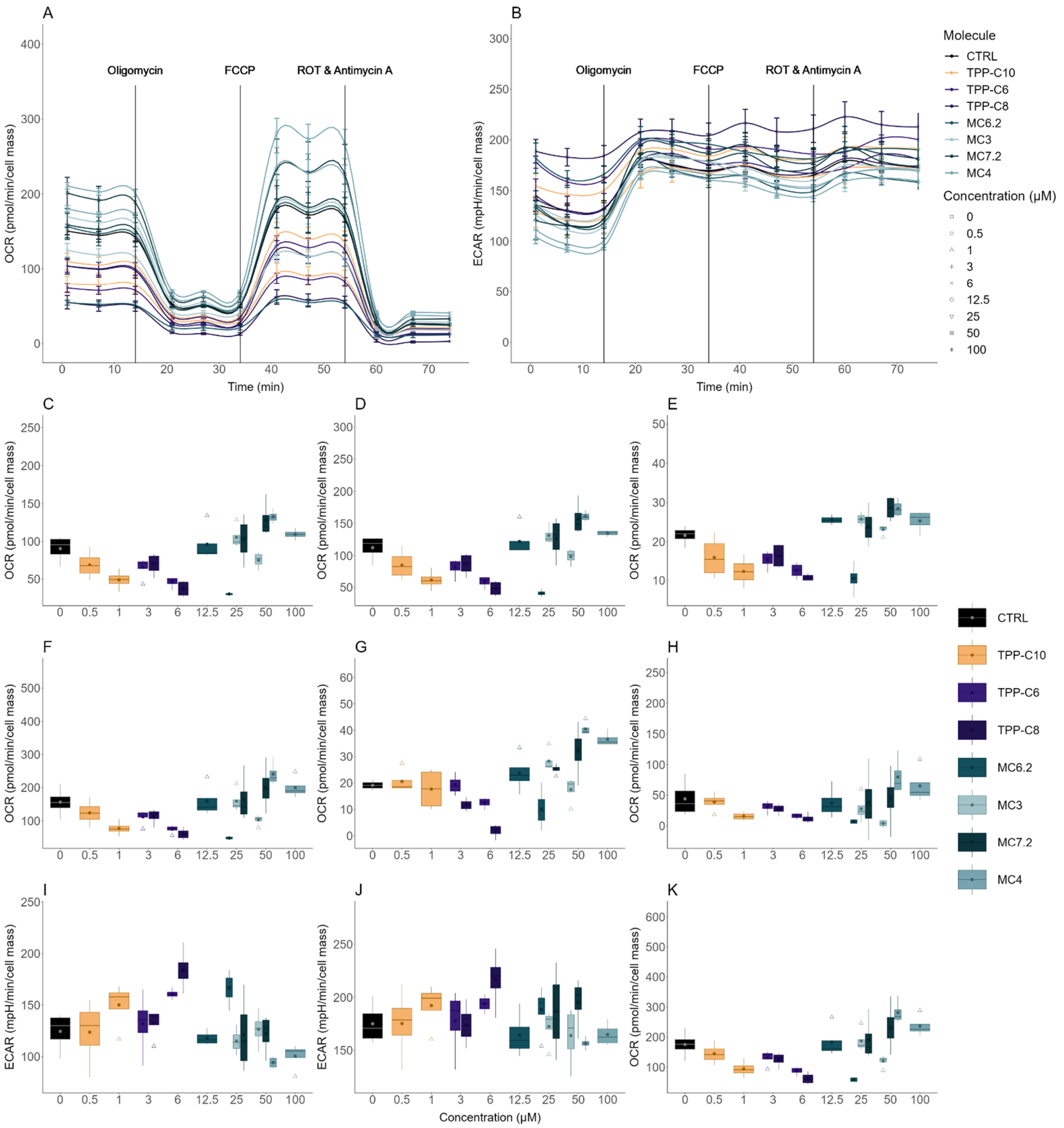


**Figure S16** Comparison between the effects of MitoCINs and alkylTPP compounds on oxygen consumption rate (OCR) and extracellular acidification rate (ECAR)in differentiated human neuroblastoma (SH-SY5Y) cells. OCR- and ECAR-associated parameters were assessed with the Seahorse XFe96 Extracellular Flux Analyzer. OCR (A)

and ECAR (B) were assessed in SH-SY5Y cells treated with increasing concentrations of the different molecules for 48 h. Several OCR parameters were evaluated: ATP production-linked OCR (C), basal respiration (D), proton leak-based OCR (E), maximal respiration (F), non-mitochondrial respiration (G), spare respiratory capacity (H) and ECAR parameters were also evaluated, including basal ECAR (I), stressed ECAR (J) and stressed OCR (K). Data are the mean± SE of four independent experiments and the results are expressed in interquartile range (Q1-Q3) together with the (–) median. Statistically significant differences between treated groups were evaluated as described in the Materials and Methods. For the sake of simplifying the figure, the different statistical comparisons in this figure are present in Tables 87-104 in the external document file (linK: https://bit.ly/3mNTtsz).

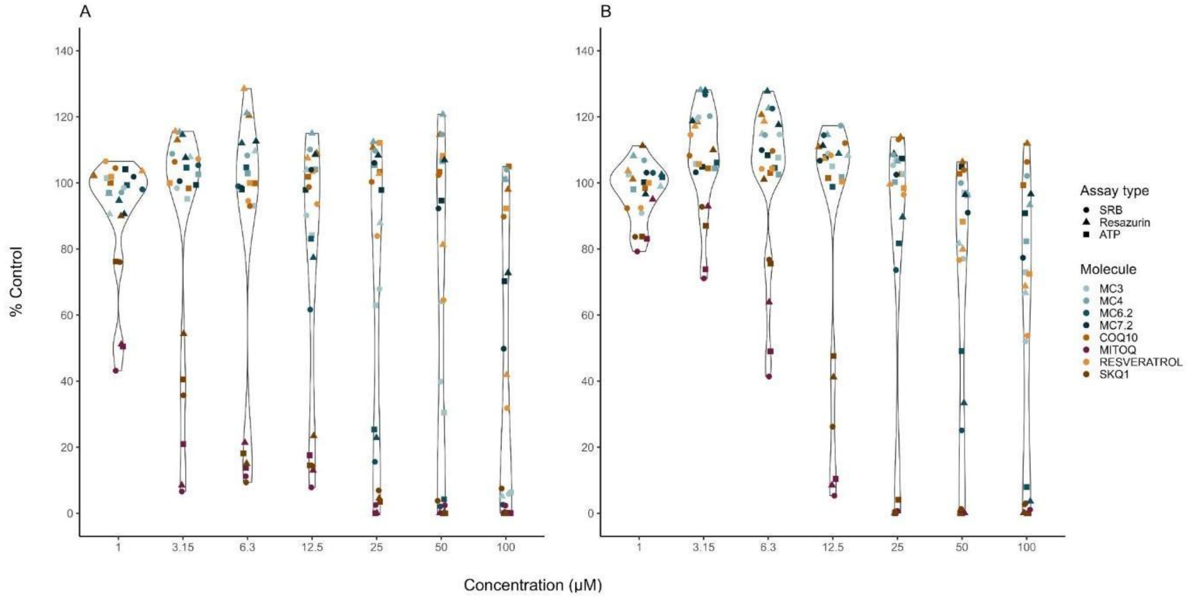


**Figure S17** Violin plot distributions of the average experimental points for HepG2 (panel A) and SH-SY5Y (panel B) viability, namey, cell mass (SRB), metabolic activity (resazurin) and ATP, under different compound treatments (MitoCINs, quinone-based mitochondria-targeted and non-targeted antioxidants) grouped by similar concentration.
